# Modulation of Asymmetric Flux in Heterotypic Gap Junctions by Pore Shape, Particle Size and Charge

**DOI:** 10.3389/fphys.2017.00206

**Published:** 2017-04-06

**Authors:** Abhijit Mondal, Frank B. Sachse, Alonso P. Moreno

**Affiliations:** ^1^Department of Bioengineering, University of UtahSalt Lake City, UT, USA; ^2^Nora Eccles Harrison Cardiovascular Research and Training Institute, University of UtahSalt Lake City, UT, USA; ^3^Department of Internal Medicine, Cardiology, University of UtahSalt Lake City, UT, USA

**Keywords:** mathematical modeling, diffusion simulation, intercellular communication, Brownian dynamics, heterotypic gap junctions, permeability

## Abstract

Gap junction channels play a vital role in intercellular communication by connecting cytoplasm of adjoined cells through arrays of channel-pores formed at the common membrane junction. Their structure and properties vary depending on the connexin isoform(s) involved in forming the full gap junction channel. Lack of information on the molecular structure of gap junction channels has limited the development of computational tools for single channel studies. Currently, we rely on cumbersome experimental techniques that have limited capabilities. We have earlier reported a simplified Brownian dynamics gap junction pore model and demonstrated that variations in pore shape at the single channel level can explain some of the differences in permeability of heterotypic channels observed in *in vitro* experiments. Based on this computational model, we designed simulations to study the influence of pore shape, particle size and charge in homotypic and heterotypic pores. We simulated dye diffusion under whole cell voltage clamping. Our simulation studies with pore shape variations revealed a pore shape with maximal flux asymmetry in a heterotypic pore. We identified pore shape profiles that match the *in silico* flux asymmetry results to the *in vitro* results of homotypic and heterotypic gap junction formed out of Cx43 and Cx45. Our simulation results indicate that the channel's pore-shape established flux asymmetry and that flux asymmetry is primarily regulated by the sizes of the conical and/or cylindrical mouths at each end of the pore. Within the set range of particle size and charge, flux asymmetry was found to be independent of particle size and directly proportional to charge magnitude. While particle charge was vital to creating flux asymmetry, charge magnitude only scaled the observed flux asymmetry. Our studies identified the key factors that help predict asymmetry. Finally, we suggest the role of such flux asymmetry in creating concentration imbalances of messenger molecules in cardiomyocytes. We also assess the potency of fibroblasts in aggravating such imbalances through Cx43-Cx45 heterotypic channels in fibrotic heart tissue.

## Introduction

Gap junction channels provide pores through cell membranes between two adjoined cells, allowing transport of cytoplasmic molecules amongst them. Each gap junction channel is formed of two coaxially placed connexons (or hemichannels) with one in each cell membrane. Each of these connexons are made of 6 circumferentially arranged connexins (Cx). To date, 21 different Cx types have been identified in humans (Söhl and Willecke, 2004). Gap junction channels are called homotypic when Cx in both hemichannels are made of the same type, and heterotypic if the Cx in each hemichannel is different. They can also be heteromeric or hetero-multimeric where multiple Cx types constitute a hemichannel. Gap junctions play key roles in intercellular communication in different tissues like the skin, nervous system, respiratory epithelium, bones, lens and retina of the eye, inner ear, heart, vasculature, and reproductive systems. Density and regulation of gap junctions are essential for cellular and tissue physiology. How these channels are relevant in the development of several diseases has become clearer as more regulatory mechanisms that involve channel expression, localization and cellular modifications are known (Spray and Dermietzel, 1995; Kelsell et al., 1997; Xia et al., 1998; Jongsma and Wilders, 2000; Uhlenberg et al., 2004).

Currently, x-ray crystallographic structures of only Cx26 and Cx43 are available (Unger et al., 1999; Maeda et al., 2009; Bennett et al., 2016), with data on molecular structure and in-pore charge distribution available only for Cx26. Structural details are a requisite for using existing computational approaches for studying membrane channels like ion channels (Allen et al., 1999; Corry et al., 2001; Chung et al., 2002; Vora et al., 2008) and porins (Schirmer and Phale, 1999; Im and Roux, 2002; Lee et al., 2011). Molecular modeling of Cx26 hemichannels (Kwon et al., 2011; Zonta et al., 2014) and gap junctions (Bennett et al., 2016; Escalona et al., 2016) was performed for studying their structure/function relations. Limitations and difficulties in deriving such structures pose a barrier to studying the structure and permeability of the whole range of gap junction channel types. Thus, most studies rely heavily on *in vitro* experimental methods like dye diffusion, double whole cell voltage and patch clamping. Fluorescent dyes were a vital tool in such studies with Lucifer yellow (LY) being the most commonly used for dye diffusion studies (Verselis et al., 1986; Cao et al., 1998; Martinez et al., 2002; Valiunas et al., 2002; Beltramello et al., 2003; Dong et al., 2006; Eckert, 2006; Rackauskas et al., 2007; Yum et al., 2007). Gap junctions serve as conduits for intercellular diffusion and transport studies of physiologically relevant molecules and ions were also conducted (Verselis et al., 1986; Veenstra et al., 1994; Goldberg et al., 1999, 2002; Niessen et al., 2000; Beyer et al., 2001; Veenstra, 2001; Martinez et al., 2002; Bedner et al., 2003, 2006; Locke et al., 2004; Valiunas et al., 2005; Ayad et al., 2006; Bukauskas et al., 2006; Harris, 2007). Our research focused on homotypic and heterotypic gap junction channels involving Cx43 and Cx45, which are abundantly present in the heart (van Kempen et al., 1995; Vozzi et al., 1999; Severs et al., 2001). In previous works, we found that Cx43 and Cx45 heterotypic channels do not exhibit electric current rectification beyond rectification caused by voltage dependent gating, which is significantly different for each connexon (Elenes et al., 2001). Calculations of dye diffusion using cell pairs under double whole cell voltage clamp experiments indicate that homotypic Cx43 channels allow higher LY flux than Cx45 channels, while heterotypic Cx43-Cx45 channels show asymmetric fluxes (Martinez et al., 2002; Moreno et al., 2004; Mondal et al., 2017). Interestingly, the recorded fluxes were higher in Cx45 to Cx43 direction than Cx43 to Cx45. Based solely on their respective unitary conductance, it was theorized that Cx43 would be a wider hemichannel than Cx45, which explained the difference in homotypic fluxes, but failed to explain the flux difference in the heterotypic Cx43-Cx45 junctions. We speculated on a structural basis for the mechanism creating this asymmetry, which was also proposed before to explain similar findings (Robinson et al., 1993). The proposed hypothesis was criticized at the time as it implied the presence of an active mechanism (Finkelstein, 1994) and found to be inconsistent with unitary conductance and permeability values from *in vitro* experiments (Veenstra et al., 1995; Veenstra, 1996). Subsequently, using a simplified Brownian dynamics computational model, we showed that a heterotypic pore can generate such an active mechanism by creating electrical fields across the pore through motion and redistribution of charged particles caused by the pore's shape (Mondal et al., 2017). The combination of a conical and cylindrical hemichannel was found to be a requisite for the underlying mechanism.

Here we use the simplified Brownian dynamics model of a gap junction pore presented in our previous work to mimic LY diffusion experiments under whole cell voltage clamp. We designed our studies to elucidate the roles of pore shape, particle size and charge on flux and flux asymmetry of large molecules. Simulation studies were conducted for both homotypic and heterotypic pores. Our studies revealed a heterotypic pore profile with maximum flux asymmetry and also pore-shapes that matched our previously reported *in vitro* results with Cx43 and Cx45 pores. We also found that within the set limits, flux asymmetry was almost independent of particle size and scaled by particle charge. While it was not possible to establish a direct relationship between pore-shape, particle charge and size, we identified factors for predicting flux asymmetry. We also speculated on a possible role of such asymmetric fluxes in heart disease.

## Methods

### Simulation model

**Figure 1:**
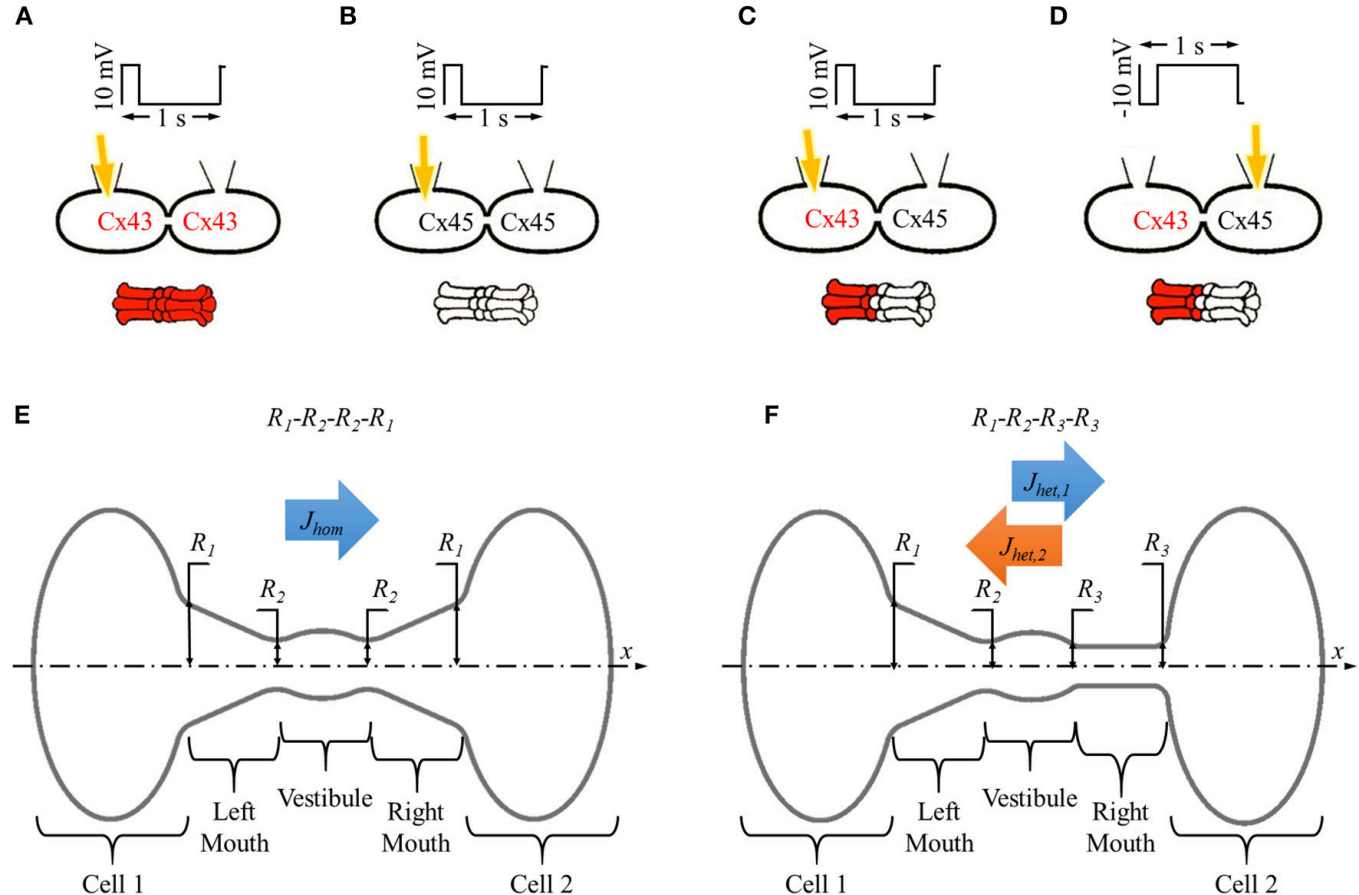
***In vitro*** **and *in silico* experiment designs with homotypic and heterotypic gap junction pores. (A–D)** Basic setup for *in vitro* dye diffusion experiments under double whole cell voltage clamp with homotypic **(A)** Cx43 and **(B)** Cx45 gap junction pores and **(C,D)** heterotypic Cx43-Cx45 gap junction pores with LY (yellow arrow) injected from **(C)** Cx43 side and **(D)** Cx45 side. Voltage pulses of 10 mV/200 ms/1 s were applied and LY dye diffusion was recorded across the gap junction. **(E,F)** Simulation experiment design for single **(E)** homotypic and **(F)** heterotypic gap junction pore. The cell-pore-cell system was divided in 5 sections: (1) cell 1, (2) left mouth, (3) vestibule (4), right mouth, and (5) cell 2.

Our simplified Brownian dynamic gap junction model (Mondal et al., 2017) was developed using MATLAB (Mathworks Inc., Natick, MA). In short, particles were modeled as spheres with point charges at their center. Stoke's radius values were adjusted for different particles size. The system was charge balanced by adding appropriate counter-ions. The simulation parameters were set to mimick *in vitro* dye diffusion under double whole cell voltage clamp experiments with coupled cells (Figures 1A–D). We modeled all our pore shape studies with Lucifer yellow (yellow arrows in Figures 1A–D) particles and Cesium (Cs) counter-ions with a 2 mV transjunctional voltage. A 2 mV transjunctional voltage would give a flux equivalent to one from 10 mV/200 ms/1 Hz pulses. A constant concentration gradient of 2 LY and 4 Cs (8 mM LY) was maintained across the pore by replacing any particle that crossed over to cell 2 (central plane of cell 2) back to cell 1. A time step of 1 ps was set. The program saved particles' positions every 10^th^ time step (10 ps). Data files containing particle positions were saved every 10 μs. The LY particle flux was calculated every 10 μs considering the total number of particles and simulation time. The simulation was terminated when the last 5 calculated LY flux values had a standard deviation <5% or simulation total time was 500 μs.

### Simulation experiment design

The simulations were run on 4 Apple Mac Pro computers involving a total of 44 (12 × 3 + 8) processing cores. Our simulation studies were designed to assess the role of pore shape, particle size, and particle charge on flux asymmetry in heterotypic pores. Simulation conditions were defined accordingly as described below.

The averaged gap junction pore was divided into 3 sections defined by 4 radii (*R*_1_, *R*_2_, *R*_3_, and *R*_4_) at the section ends placed in series. A pore was identified by listing these pore-section radii (in Å) in order from left to right as *R*_1_-*R*_2_-*R*_3_-*R*_4_. Homotypic pores were symmetric about the vestibule, *R*_1_ = *R*_4_ and *R*_2_ = *R*_3_ (Figure 1E) and were denoted as *R*_1_-*R*_2_-*R*_2_-*R*_1_. Heterotypic pores studied in this work were always in a cone-cylinder combination of hemichannels, which meant *R*_3_ = *R*_4_. This was a required condition for activating the mechanism that resulted in asymmetric fluxes in heterotypic pores (Mondal et al., 2017). So, in all our simulations, heterotypic pores will be of the form *R*_1_-*R*_2_-*R*_3_-*R*_3_. A range of values were set for the outer (*R*_1_) and inner pore (*R*_2_) radii taking into consideration the particle size and computation time. In our studies, inner mouth radii ranged from 6.2 to 12.2 Å and outer mouth radii ranged from 6.2 to 25.9 Å. In all our pore radii combination, outer mouth radii were never smaller than the inner mouth radii to avoid inverted conical pore sections.

#### Flux simulations in homotypic pores (*R*_1_-*R*_2_-*R*_2_-*R*_1_)

Table 1 lists the inner (*R*_1_) and outer (*R*_2_) mouth radii considered for forming homotypic pores. Figure 1E shows the homotypic pore simulation setup. Based on our set criteria for selecting combinations, 56 homotypic pores were considered. Flux simulations were run for each pore 30 times (*n* = 30).

**Table 1:** **Sectional radii of homotypic pores**.

*R*_1_ (Å)	6.2, 7.2, 8.2, 9.2, 10.2, 11.2, 12.2, 15.8, 20.2, 22.5, 25.9
*R*_2_ (Å)	6.2, 7.2, 8.2, 9.2, 10.2, 11.2, 12.2

#### Flux simulations in heterotypic pores

Our previous work (Mondal et al., 2017) was focused around the hypothesis that flux asymmetry in heterotypic pores can be produced if one of the pore mouths was cylindrical and the other was conical. Simulation were designed to study the effects of *R*_1_, *R*_2_, and *R*_3_ in different simulation sets (rows 1–3 in Table 2). Each simulation case was repeated 50 times (*n* = 50) on either direction (cone to cylinder and cylinder to cone) of the heterotypic pore. In the 1^st^ set of simulations (row 1 in Table 2), *R*_1_ was varied while *R*_2_ and *R*_3_ were fixed to 9.2 Å. This basically simulates the effect of varying cone angle of the conical mouth of the pore. In the 2^nd^ set of simulations, *R*_2_ was varied, with *R*_1_ = 22.5 Å and *R*_3_ = 9.2 Å. Here, the influence of inner mouth size was studied. In the 3^rd^ set, the cylinder mouth size was varied while the left conical mouth was kept constant, with *R*_1_ = 22.5 Å and *R*_2_ = 9.2 Å. Finally, in our 4^th^ simulation set, the conical mouth was set to the maximal *R*_1_ and *R*_2_, with *R*_1_ = 25.9 Å and *R*_2_ = 12.2 Å and the effects of varying the cylinder mouth were investigated.

**Table 2:** **Sectional radii combinations for heterotypic pores**.

**S#**	**Shape combination (Å)**	**Radius (Å)**
1	*R*_1_-9.2-9.2-9.2	*R*_1_ = {9.2, 10.2, 11.2, 12.2, 15.8, 20.2, 22.5, 25.9}
2	22.5-*R*_2_-9.2-9.2	*R*_2_ = {6.2, 7.2, 8.2, 9.2, 10.2, 11.2, 12.2}
3	22.5-9.2-*R*_3_-*R*_3_	*R*_3_ = {6.2, 7.2, 8.2, 9.2, 10.2, 11.2, 12.2}
4	25.9-12.2-*R*_3_-*R*_3_	*R*_3_ = {6.2, 7.2, 8.2, 9.2, 10.2, 11.2, 12.2}

#### Flux simulations with varying particle size

In this set of simulations, the particle size was varied from 0.8 to 6.6 Å. Row 1 in Table 3 lists the heterotypic pore and the particle radii values used. While the particle size was varied, the counter-ion size was kept constant. Particle charge was set to 2*e*^−^.

**Table 3:** **Particle sizes and charges for heterotypic pore simulations**.

**S#**	**Pore profile (Å)**	**Parameter**	**Values**
1	25.9-12.2-8.2-8.2	Particle radius, *r_*p*_* (Å)	*r_*p*_* = {0.8, 1.79, 2.7, 3.8, 4.9, 6, 6.6}
2	25.9-12.2-8.2-8.2	Particle charge, *q_*p*_* (*e*^−^)	*q_*p*_* = {0, 0.5, 1, 2, 2.5}

#### Flux simulations with varying particle charge

In our previous work, particle charge was an important factor in creating the observed flux asymmetry of large ionic particles. In this set of simulations, we varied the particle charge (Row 2 in Table 3) to study the influence of charge on flux asymmetry. The number of counter-ions was accordingly changed to maintain charge balance. Particle radii of LY and Cs were not changed.

### Simulation output and analysis

Simulation data were collected and processed for analysis on an 8 core Apple Mac Pro computer using MATLAB R2016a. This included data verification, tabulating particle fluxes, calculating particle probabilities and sectional forces and data organization. The final result tables and plots were created with Excel 2013 (Microsoft Inc., Redmond, WA).

#### Particle flux and flux asymmetry

Particle flux *J* was calculated by counting the total number of particles that crossed the pore *n*_*p*_ divided by the total simulation time *t*.

(1) $$J=\frac{n_{p}}{t}$$

In homotypic pores, flux was only measured in the *x* direction where the concentration gradient of 2LY + 4Cs to 0 particles was maintained between cell 1 and cell 2 and denoted by *J*_*hom*_ (Figure 1E). In heterotypic pores, flux was measured in the *x* direction with 2LY + 4Cs to 0 particles in cell 1 to cell 2 (or cone to cylinder direction), and the -*x* direction with 2LY + 4Cs to 0 particles in cell 2 to cell 1 (or cylinder to cone direction) and denoted by *J*_*het*_,_1_ and *J*_*het*_,_2_, respectively (Figure 1F).

Flux ratio was the measure of flux asymmetry α_*het*_ in a heterotypic pore and defined as the ratio of the LY (or particle) fluxes in cone to cylinder (*J*_*het*_,_1_) and cylinder to cone (*J*_*het*_,_2_) directions.

(2) $$\alpha_{het}=\frac{J_{het,1}}{J_{het,2}}$$

Heterotypic flux ratios were plotted in log scale (base 2) to accurately scale the magnitude of flux asymmetry in reversed cases (e.g., α_*het*_ of 0.5 and 2 are equal in magnitude but in opposite directions).

Ratio of the homotypic fluxes of the corresponding hemichannels in a heterotypic pore α_*hom*_ was also calculated to identify reversal of flux asymmetry.

(3) $$\alpha_{hom}=\frac{J_{hom,1}}{J_{hom,2}}$$

where *J*_*hom*_,_1_ and *J*_*hom*_,_2_ are the fluxes in corresponding homotypic pores formed of the left and right hemichannels, respectively.

#### Particle probability

Particle probability profiles along the pore's axial profile (along *x*-axis) were calculated from the simulation data and plotted. Axial particle probability *P*_*dX*_ was the probability of particle presence within a cylindrical section of width *dX* at **x** of the pore:

(4) $$\begin{array}{l} \;P_{dx}=\frac{\Delta t}{t_{total}}\sum_{i\;=\;1}^{n_{p}}\sum_{n\;=\;1}^{n_{t}}p_{dx}(i,n)\\ where\;p_{dX}\left(i,n\right)=\{\begin{array}{c} 1,\;x\left(i,n\right)\;lies\text{ within }dX\;\\ 0,\text{ otherwise} \end{array} \end{array}$$

Here, Δ*t* is the time step, *t*_*total*_ is the total simulation time, *N*_*p*_ is the number of particles, *N*_*t*_ is the number of time steps, **x** is the particle position, and *i* and *n* are the particle and time indexes, respectively.

#### Average electrical force per particle per pore section

The average electrical force per particle per pore section was calculated as in our previous work (Mondal et al., 2017). In short, the pore was divided into 5 sections in series: (1) cell 1 (C1), (2) left mouth (LM), (3) vestibule (VT), (4) right mouth (RM), and (5) cell 2 (C2) as shown in Figures 1E,F). The average interparticle electrostatic and electric field forces per particle type **F** was calculated. The x-component of **F** F_X_ was plotted and compared amongst the different simulation conditions.

While the actual simulations were done in +*x* and −*x* directions, the −*x* data for *P*_*dX*_ and F_X_ data presented here were flipped horizontally so all flux direction are in the +*x* direction. This simplified it for the reader to compare the presented data in the different cases.

## Results

The results presented in the next sections were derived from ~2.2 TB simulation data resulting from ~28.3 days of computation time per processing core (44 processors). This excludes data handling and postprocessing done on the central workstation (8 processing cores).

### Flux simulations in homotypic pores

Fluxes across homotypic pores do not present asymmetry. Table S1 list the fluxes for all 56 homotypic pores considered. Figures S1–S4 present LY fluxes, particle probability profiles and average electrical force data arranged to study variations in outer (*R*_1_) and inner (*R*_2_) mouth radii. LY flux values in our simulation with 8 to 0 mM LY gradient ranged from 3,551 molecules per pore per s (m/c/s) to 64,588 m/c/s in homotypic pores (Table S1) and 2,919 m/c/s to 51,409 m/c/s in heterotypic pores (Table S2). Physiological reported values of LY flux range from 4,000 m/c/s (m/c/s) (Valiunas et al., 2002; Ek-Vitorín and Burt, 2005; Ek-Vitorín et al., 2006; Heyman and Burt, 2008; Kanaporis et al., 2013) to 300,000 m/c/s (Weber et al., 2004).

#### Outer mouth variation in conical mouthed pores: *R*_1_-6.2-6.2-*R*_1_ and *R*_1_-12.2-12.2-*R*_1_

Results for fixed inner mouth radii (*R*_2_) of 6.2 Å (narrow mouth) and 12.2 Å (wide mouth) with varying outer mouth radius are presented in Figures S1, S2, respectively. In pores with narrow inner mouth (*R*_2_ = 6.2 Å), an increasing outer mouth marginally changed the LY flux (Figure S1A). When the inner mouth radius was widened (*R*_2_ = 12.2 Å), increasing the outer mouth radius increased the LY flux which tended to saturate at higher values of *R*_1_ (Figure S2A).

The *P*_*dX*_ profile in cell 1 was of the same order in all cases (Figures S1B, S3B) indicating a constant LY concentration in cell 1. As the outer mouth was varied, the *P*_*dX*_ profile varied across the pore. The vestibule size was kept constant and for wide inner pore mouth (*R*_2_ = 12.2 Å), the *P*_*dX*_ profiles in the vestibule were of the same order. The *P*_*dX*_ profiles tended to be noisier with increasing distance along *x* from cell 1, and were noisiest at the rightmost region (cell 2). In general, the particle probability profiles were reminiscent of their expected concentration profiles based on the pore's radius profile in simple diffusion, but in the logarithmic scale (base 10). *R*_2_ = 6.2 Å created probability profiles with the most variations along the pore sections, while *R*_2_ = 12.2 Å presented a more gentle probability variation.

Homotypic pores with *R*_2_ = 6.2 Å had the highest F_X_ magnitudes (Figures S1C, S3C). These were mostly observed in the left and right mouth of the pore. F_X_ also varied the most in those sections with varying outer mouth sizes. In the left mouth though, the trend was nonlinear. F_X_ rose in magnitude up to 8.2 Å and then dropped as outer mouth size was further increased to 25.9 Å.

The case *R*_1_ = *R*_2_ = 6.2 Å showed highest F_X_ magnitudes in the right mouth (Figure S1C). F_X_ decreased as the outer mouth size was increased. In pores with *R*_2_ = 12.2 Å (Figure S2C), F_X_ showed less variation than that in *R*_2_ = 6.2 Å. A narrow inner mouth caused more variations in F_X_ in the left and right mouths (Figure S1C) while a wide mouth created notable F_X_ variations in the vestibule (Figure S2C). Furthermore, in narrow mouthed pores, F_X_ in the left mouth rose up to *R*_1_ = 15.8 Å and then dropped with increasing *R*_1_. In the wide mouthed pore, F_X_ in the vestibule had almost a linear rise with increasing *R*_1_.

#### Inner mouth variation in conical mouthed pores (22.5-*R*_2_-*R*_2_-22.5)

Simulation data were selected and arranged for *R*_1_ = 22.5 Å and varying inner mouth size of *R*_2_ = 6.2 Å to 12.2 Å (Figure S3). LY flux exhibited a nonlinear rise with increasing inner mouth size (Figure S3A). *P*_*dX*_ remained almost the same up to the initial section of the left mouth (Figure S3B). Pronounced variation among the varying inner mouth size conditions was observed closer to the inner mouth and to the right of that region. Variations were amplified at the inner mouth regions on both sides of the vestibule. F_X_ in the left mouth and vestibule were negative and most distinct. While F_X_ magnitude dropped with increased *R*_2_, it rose in the vestibule. Increasing *R*_2_ did not generate noticeable variations in F_X_ in cell 2.

#### Cylindrical mouthed pores (*R*_1_-*R*_2_-*R*_2_-*R*_1_)

Figure S4 summarizes results from flux simulations with cylindrical pores (*R*_1_ = *R*_2_) where the cylinder size was increased from 6.2 to 12.2 Å. The complete pore with *R*_1_ = *R*_2_ value of 12.2 Å was almost a cylinder as the elliptical vestibule almost disappears (bottom right corner of Figure S4A). LY flux rose nonlinearly with increasing pore mouth size (Figure S4A). *P*_*dX*_ profiles of the different pore sizes ran parallel in the left and right mouth sections (Figure S4B). Vestibular sections of the *P*_*dX*_ profile straightened with increase in cylinder radius. As before, the probability profile followed the pore-section in the logarithmic scale. At 12.2 Å, the particle probability was almost a straight line with no elliptical bump at the vestibule region. The left mouth and right mouth had the most pronounced F_X_ magnitudes (Figure S4C). F_X_ was negative in all cases, except in the right mouth for cylinder radius of 6.2 Å. In the left mouth, F_X_ linearly decreased with increasing cylinder radius. F_X_ magnitudes in cell 1 were marginal. In cell 2, the differences in F_X_ for different cylinder radii were marginal.

### Flux simulations in heterotypic pores

#### Varying outer mouth size of conical section (*R*_1_-9.2-9.2-9.2)

**Figure 2:**
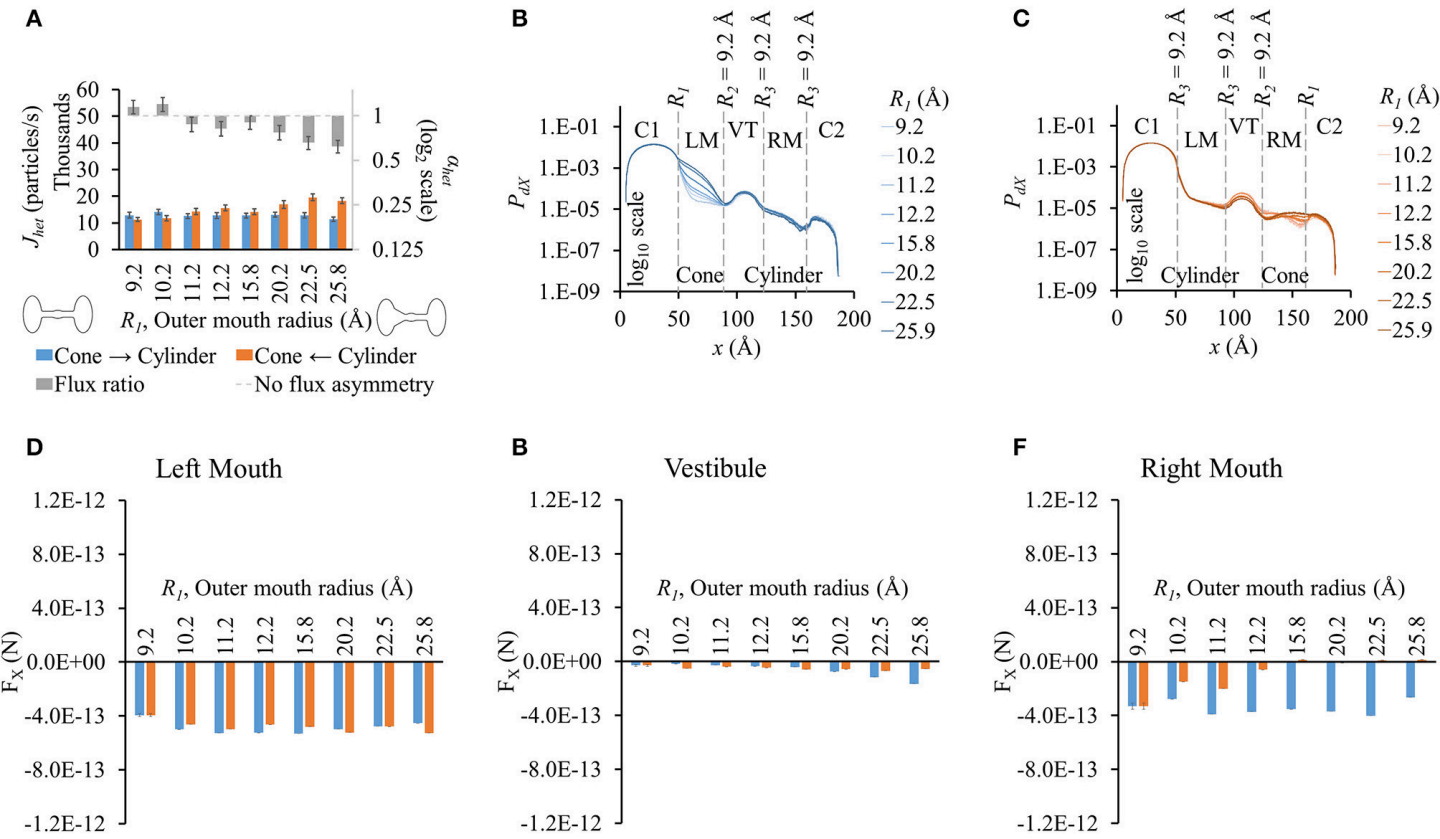
**Properties of heterotypic pores with varying outer mouth size of conical section (*R*_1_-9.2-9.2-9.2). (A)** LY fluxes and flux ratios simulated with constant concentration gradient of 2LY + 4Cs in cone to cylinder (blue bars) and cylinder to cone (orange bars) directions. The difference between *J*_*het*_,_1_ and *J*_*het*_,_2_ increased with larger conical mouth size. LY flux asymmetry increased with increasing outer mouth size of the conical section. **(B,C)** *P*_*dX*_ profiles in simulation **(B)** cone to cylinder direction and **(C)** cylinder to cone direction. **(D–F)** Average force per LY particle in **(D)** left mouth, **(E)** vestibule, and **(F)** right mouth. F_X_ was in the -*x* direction in all sections of the pore. **(D)** F_X_ magnitudes in the left mouth in either direction were almost equal for different pore mouth sizes. **(E)** F_X_ magnitudes in the vestibule rose in the cone to cylinder direction with increase in *R*_1_. **(F)** The right mouth had high differences in F_X_ magnitudes between the two directions with F_X_ magnitude dropping with increasing *R*_1_ in the cylinder to cone direction.

Increasing the outer mouth size of the conical section marginally changed LY flux in the cone to cylinder direction (blue bars in Figure 2A). However, LY flux in the cylinder to cone direction rose (orange bars in Figure 2A). This caused an increase in the difference between the fluxes in the two direction as *R*_1_ was increased. This contributed to an increase in flux ratio, which shows a gentle rise with increasing *R*_1_ (gray bars in Figure 2A). The cone with the widest mouth showed the highest asymmetry (*R*_1_ = 25.9 Å).

As in homotypic pores, the *P*_*dX*_ profiles varied primarily due to the differences in pore shapes and the differences were most prominent in the section where size was varied. The differences were most prominent in the conical mouth in either direction (Figures 2B,C). In the cone to cylinder direction simulations, *P*_*dX*_ levels in the left mouth were seen to drop in the left mouth as *R*_1_ was reduced (Figure 2B). In the remaining pore (vestibule and right mouth), the *P*_*dX*_ profiles were of the same shape and in the same order of magnitude. In the cylinder to cone direction simulations (Figure 2C), the *P*_*dX*_ levels began to notably vary from the end of the left mouth (end of conical mouth) itself. Particle probabilities were higher in the vestibule for smaller values of *R*_1_ and declined as *R*_1_ was increased. This trend was reversed in the center region of the right mouth (right mouth section in Figure 2C).

As in homotypic pores, F_X_ in cell 1 was marginal compared to F_X_ in other pore sections (Figures S5A–E). The left (Figure 2D) and right (Figure 2F) mouths had high F_X_ values. F_X_ values were negative throughout the pore in simulations of both direction. The left mouth F_X_ differences between either directions were marginal for all cases. F_X_ in the vestibule (Figure 2D), though small in magnitude, showed a widening difference between the 2 directional fluxes as *R*_1_ was increased with F_X_ in the cone to cylinder direction being larger (Figure 2E). Differences in F_X_ magnitudes were high in the right mouth (Figure 2F).

#### Varying inner mouth size of conical section (22.5-*R*_2_-9.2-9.2)

**Figure 3:**
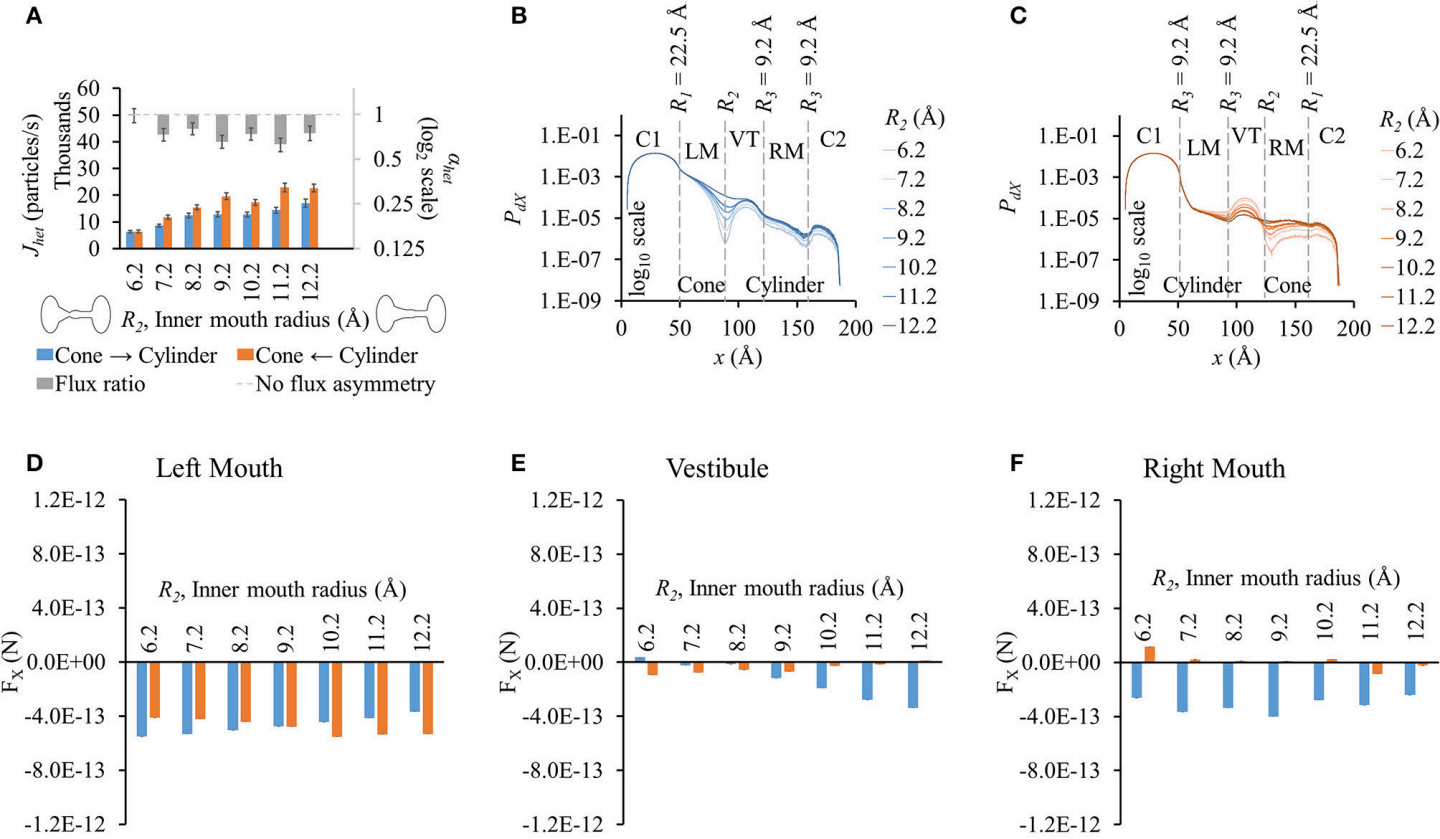
**Properties of heterotypic pores with varying inner mouth size of conical section (22.5-*R*_2_-9.2-9.2). (A)** LY fluxes rose in both directions with increasing inner mouth size (only *R*_2_). Differences between *J*_*het*_,_1_ and *J*_*het*_,_2_ vary amongst the different cases. LY flux ratio variations appeared to be multimodal with increasing *R*_2_ with maximum flux asymmetry at *R*_2_ = 9.2 Å and 11.2 Å. **(B)** *P*_*dX*_ in cone to cylinder direction dropped near *R*_2_ and maintained almost a uniform difference amongst the different inner pore mouth sizes. **(C)** *P*_*dX*_ in the cylinder to cone direction decreased in the left mouth and vestibule with increasing *R*_2_. This tendency flipped by the end of the vestibule with *P*_*dX*_ levels increased with increase in *R*_2_ in the right mouth. **(D–F)** Average force per LY particle in **(D)** left mouth, **(E)** vestibule, and **(F)** right mouth. **(D)** Increase in *R*_2_ caused F_X_ magnitudes (-*x* direction) in the left mouth to gently decrease in the cone to cylinder direction, and gently rise in the cylinder to cone direction. **(E)** In the vestibule, F_X_ increased in the cone to cylinder direction, while decreased in the cylinder to cone direction with increase in *R*_2_. **(F)** F_X_ magnitudes in the cone to cylinder direction were negative and varied randomly in the right mouth.

Flux values rose with increasing inner mouth size in both directions (blue and orange bars in Figure 3A). Difference in flux in either direction seemed to fluctuate as *R*_2_ was increased. This variation appeared to be multimodal as observed in flux ratios which varied from ~0.7 to 1 (gray bars in Figure 3A). Due to high standard error magnitudes, a clear dependence of *R*_2_ on flux asymmetry was hard to identify with maximum flux asymmetry at *R*_2_ = 9.2 Å and 11.2 Å and no flux asymmetry seen at *R*_2_ = 6.2 Å.

Particle probability profiles pointed to the vestibular mouth (at *R*_2_ or end of conical mouth) as the main source of *P*_*dX*_ variation in the cone to cylinder direction (Figure 3B). This order of difference in magnitude due to varying *R*_2_ was maintained in the remaining pore profile. The *P*_*dX*_ profiles in the cylinder to cone direction exhibited different variations (Figure 3C). While *P*_*dX*_ remained in the same order up to the vestibule mouth (or end of the cylinder mouth), it was highest for *R*_2_ = 6.2 Å and lowest for *R*_2_ = 12.2 Å in the vestibule region. By the end of the vestibule (smaller end of the conical mouth), this trend was inversed and *P*_*dX*_ dropped along *x* with lowest levels at *R*_2_ = 6.2 Å and highest at *R*_2_ = 12.2 Å in the region and the remaining right mouth (conical section).

F_X_ differences between the two directions were pronounced in the vestibule (Figure 3E) and right mouth (Figure 3F) with higher F_X_ against the flux (negative) in the cone to cylinder direction. This rose in the cone to cylinder direction with increasing *R*_2_ in the vestibule (blue bars in Figure 3E). The variation in F_X_ in the right mouth was random. In the left mouth, F_X_ magnitude (in -*x* direction) in the cone to cylinder direction fell, while rising in the cylinder to cone direction as *R*_2_ was increased (Figure 3D). In general, the net electrical force acting against the direction of flux was more in the cone to cylinder direction than in the cylinder to cone direction.

#### Varying cylindrical mouth size (22.5-9.2-*R*_3_-*R*_3_)

**Figure 4:**
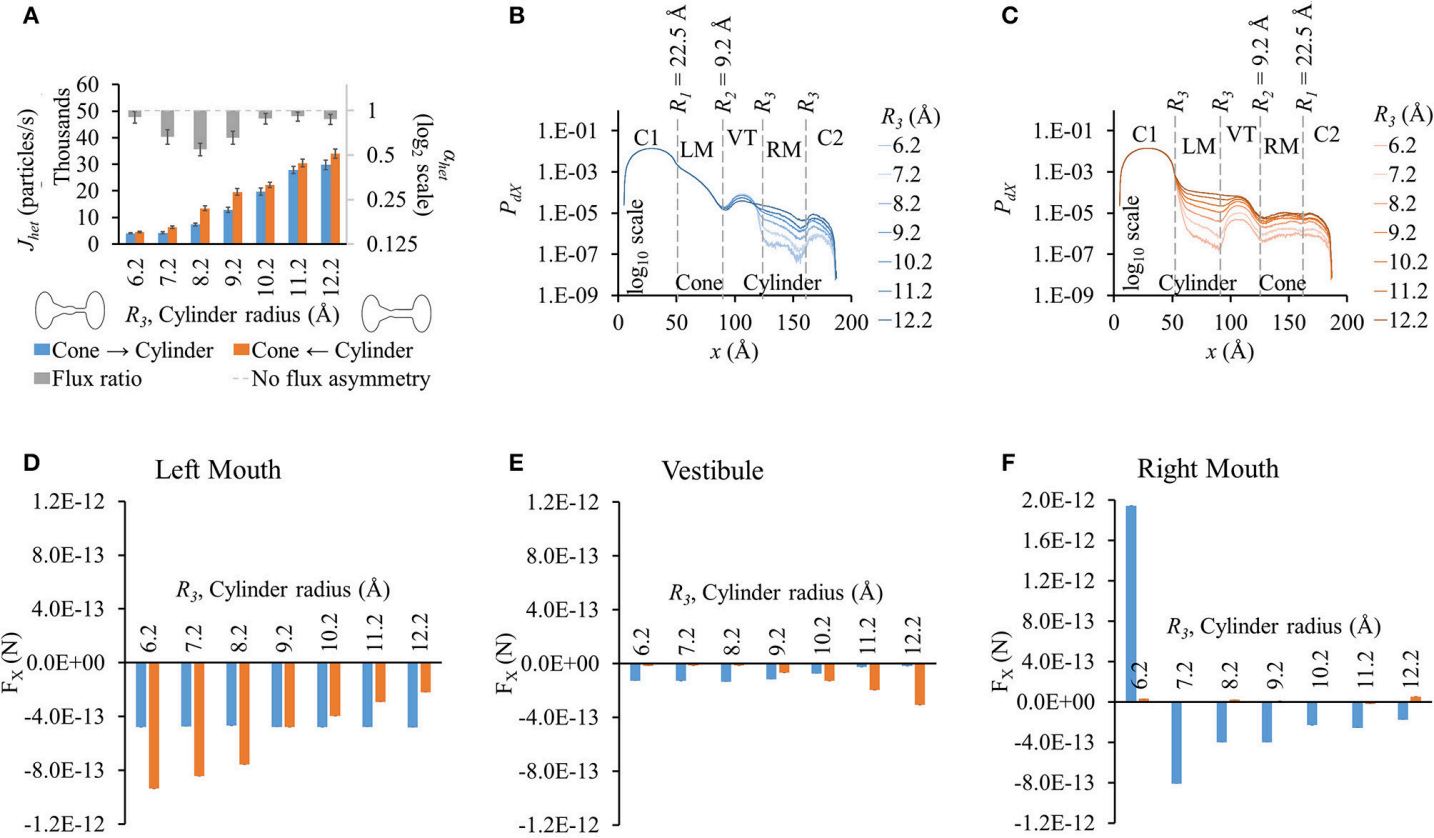
**Properties of heterotypic pores with varying cylindrical mouth size (22.5-9.2-*R*_3_-*R*_3_). (A)** LY fluxes increased in both directions as the cylinder section was widened. LY flux ratio rose and then dropped with increasing cylinder mouth size with maximal at *R*_3_ = 8.2 Å. **(B)** *P*_*dX*_ levels remained in the same order up to the mouth of the vestibule in cone to cylinder direction for the different cylinder mouth sizes. Probability profiles almost ran parallel from there on for all cases with probability levels proportional to *R*_3_. **(C)** *P*_*dX*_ levels dropped at the beginning of the cylindrical mouth (left mouth) and mimicked the pore profile up to the vestibule, after which they gently rose. Profiles for different cylinder sizes ran almost parallel. **(D–F)** Average force per LY particle in **(D)** left mouth, **(E)** vestibule, and **(F)** right mouth. **(E)** In the cone to cylinder direction, F_X_ in the left mouth was marginally affected by increase in the cylinder mouth size. In the cylinder to cone direction though, F_X_ dropped as the cylindrical mouth was widened. **(E)** In the vestibule, F_X_ gently dropped in the cone to cylinder direction while rising in the cylinder to cone direction with widening cylindrical mouth. **(F)** F_X_ magnitudes in the right mouth were larger in the cone to cylinder than cylinder to cone direction with magnitude dropping with increasing *R*_3_. F_X_ was negative for all but *R*_3_ = 6.2 Å.

Fluxes rose with increased cylinder mouth size in both directions (blue and orange bars in Figure 4A). Flux ratio, on the other hand, followed a bell curve with maximal at *R*_3_ = 8.2 Å. Flux asymmetry appeared to be present only at cylinder mouth sizes between ~7.2 and 9.2 Å.

There were no noticeable differences in *P*_*dX*_ along *x* up to the vestibule (or end of the conical mouth) with varying cylinder size in the cone to cylinder direction (Figure 4B). In the vestibule, *P*_*dX*_ was inversely proportional to the cylinder radius with smaller cylinders having *P*_*dX*_ profiles an order of magnitude higher than the widest cylinder. In the right mouth, *P*_*dX*_ levels dropped gently and were proportional to the cylinder radius with *P*_*dX*_ profiles parallel to each other. On the other hand, in cylinder to cone direction simulations, *P*_*dX*_ profiles (Figure 4C) varied from the beginning of the cylindrical mouth to the end of the pore with *P*_*dX*_ values almost maintaining contoured profiles to each other with *P*_*dX*_ levels proportional to the cylinder mouth size.

F_X_ was negative or marginal in all cases except for *R*_3_ = 6.2 Å in the cylinder mouth (right mouth, Figure 4F) in the cone to cylinder direction. As *R*_3_ was increased, F_X_ dropped in the cylinder to cone direction while remaining almost constant in the cone to cylinder direction. In the vestibule, F_X_ remained constant up to *R*_3_ = 8.2 Å and then decreased with widening cylinder mouth in the cone to cylinder direction (blue bars in Figure 4E). A similar trend was seen in the cylinder to cone direction where F_X_ remained almost the same up to *R*_3_ = 8.2 Å, but then rose with widening of the cylindrical mouth (orange bars in Figure 4E). In the right mouth, while F_X_ remained marginal in the cylinder to cone direction, it dropped with increasing *R*_3_ from 7.2 to 12.2 Å (Figure 4F). We looked at differences in F_X_ amongst the two flux directions and found that the F_X_ difference reversed in the left mouth and vestibule after *R*_3_ = 9.2 Å.

#### Varying cylindrical mouth size in pore with wide conical mouth (25.9-12.2-*R*_3_-*R*_3_)

**Figure 5:**
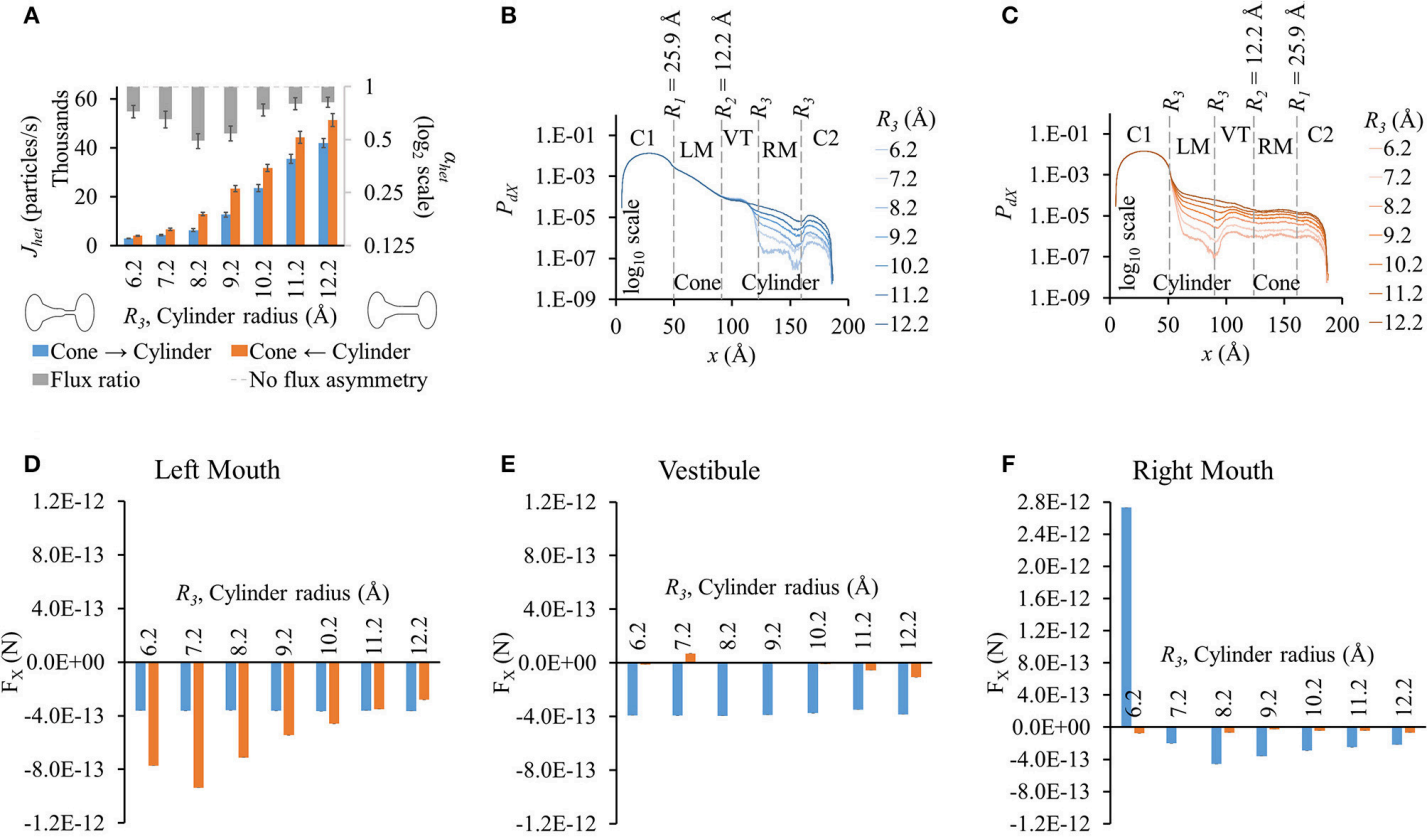
**Properties of heterotypic pores with wide conical mouth and varying cylindrical mouth size (25.9-12.2-*R*_3_-*R*_3_). (A)** LY fluxes rose in both directions with increasing cylinder size. Fluxes in cone to cylinder direction were smaller than in cylinder to cone direction for all cylindrical section sizes. LY flux ratios varied nonlinearly with cylinder mouth size with maximal at *R*_3_ = 8.2 Å. **(B,C)** *P*_*dX*_ profiles in simulations with concentration gradient in **(B)** cone to cylinder and **(C)** cylinder to cone direction. *P*_*dX*_ profile variations were similar to those seen in the 22.5-9.2-*R*_3_-*R*_3_ simulations. **(D–F)** Average force per LY particle in **(D)** left mouth, **(E)** vestibule, and **(F)** right mouth. F_X_ in the left mouth and vestibule in the cone to cylinder direction simulations remained constant with increasing cylinder size. In the left mouth **(D)**, F_X_ magnitude in cylinder to cone direction varied nonlinearly with a maximal at *R*_3_ = 7.2 Å. **(F)** F_X_ magnitudes in the right mouth were larger in the cone to cylinder than cylinder to cone direction with magnitude dropping with increasing *R*_3_. F_X_ was negative for all but *R*_3_ = 6.2 Å.

Results were similar to those with pore profile of 22.5-9.2-*R*_3_*-R*_3_ (Figure 5A). Fluxes rose with wider cylinder mouth and flux ratio was seen to be maximal at *R*_3_ = 8.2 Å. Maximum LY flux asymmetry (0.49) was slightly higher than observed in 22.5-9.2-*R*_3_*-R*_3_ simulations (0.55).

*P*_*dX*_ profiles (Figures 5B,C) and variations amongst different cylinder sizes were also similar to those for 22.5-9.2-*R*_3_*-R*_3_, with the profiles showing pronounced differences starting from the end of the vestibule in the cone to cylinder direction and beginning of the cylinder mouth in the cylinder to cone direction. Since a wider conical mouth was used, the drop in *P*_*dX*_ along *x* at the beginning and end of the conical mouth was smaller than seen in 22.5-9.2-*R*_3_*-R*_3_. (Figure 5C vs. Figure 4C).

While *P*_*dX*_ variations were similar to those in the previous pore-shape cases (22.5-9.2-*R*_3_*-R*_3_), F_X_ variations were notably different. F_X_ in the cone to cylinder direction remained almost constant in the left mouth (Figure 5D) and vestibule (Figure 5E) with increasing cylinder mouth size. In the right mouth, F_X_ was positive at *R*_3_ = 6.2 Å, then dropped to negative at *R*_3_ = 7.2 Å, reaching minimal at *R*_3_ = 8.2 Å and then rising again up to *R*_3_ = 12.2 Å (Figure 5F). On the other hand, F_X_ magnitudes in the cylinder to cone direction were marginal in the vestibule and right mouth. In the left mouth, it increased up to *R*_3_ = 7.2 Å and then dropped, while being greater in magnitude to its corresponding cone to cylinder direction up to *R*_3_ = 11.2 Å (Figure 5D). While in the left mouth, the force against the flux (negative F_X_) was higher in the cylinder to cone direction (*R*_3_ = 6.2 to 10.2 Å), it was higher in the cone to cylinder direction in the vestibule and right mouth (*R*_3_ = 7.2 to 12.2 Å).

From all different heterotypic shapes studied, a pore-profile of 25.9-12.2-8.2-8.2 yielded maximum LY flux asymmetry with flux ratio of ~0.49. Flux ratio of the corresponding homotypic pores of the left and right hemichannels was ~9.18.

### Influence of particle size on flux asymmetry in heterotypic pores

**Figure 6:**
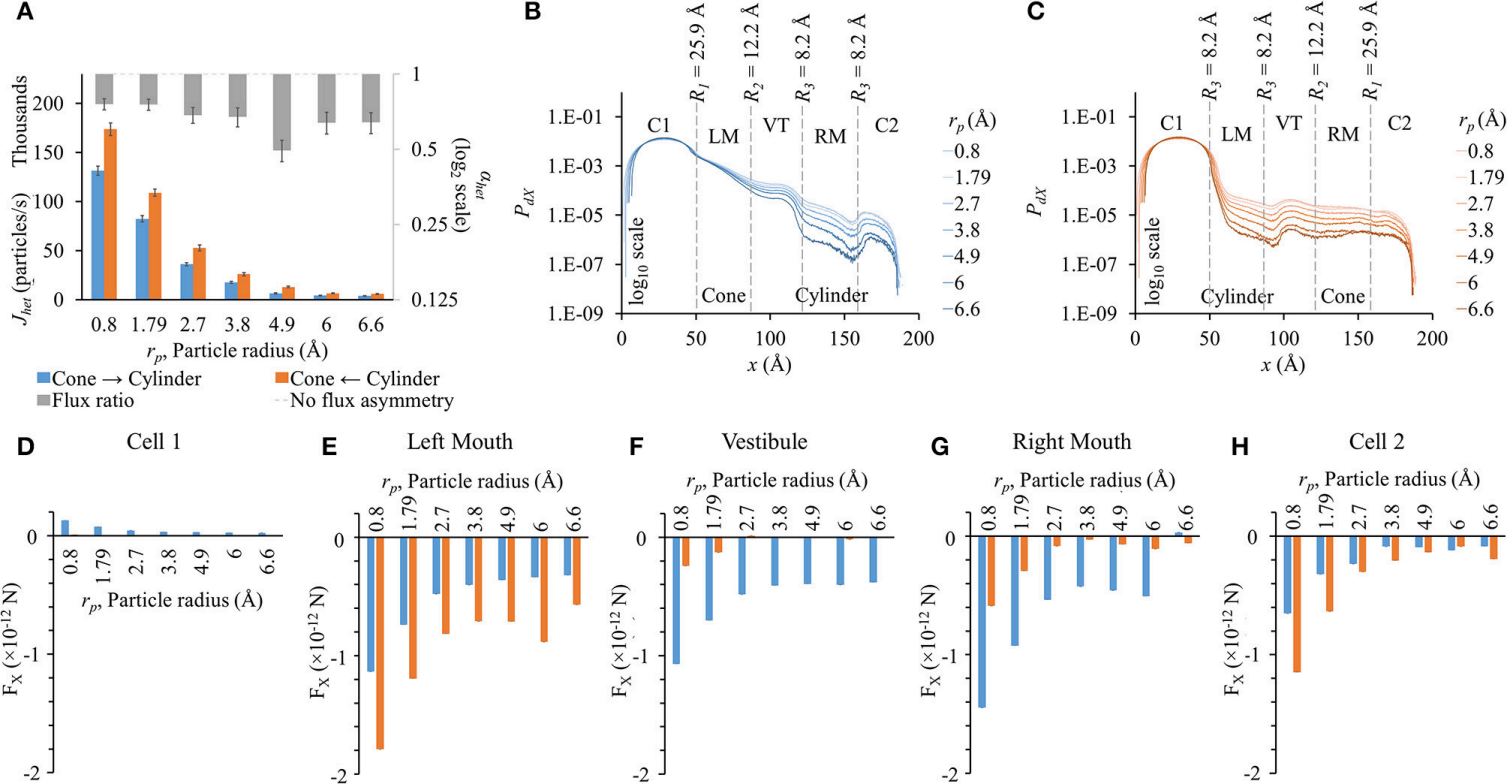
**Role of particle size on flux asymmetry in heterotypic pores**. Simulation results in heterotypic pore with profile 25.9-12.2-8.2-8.2 with constant concentration gradient, 2 mV transjunctional voltage and *q*_*p*_ = 2*e*^−^. **(A)** Particle fluxes dropped nonlinearly as particle radius was increased from 0.8 to 6.6 Å. Flux ratio values (gray bars) ranged from ~0.5 to 0.8. Maximum asymmetry of 0.51 was for particle size of 4.9 Å. **(B,C)** *P*_*dX*_ levels remain in the same order in cell 1. *P*_*dX*_ profiles were similar to the pore profiles (log 10 scale). **(D–H)** F_X_ in the different sections of the cell-pore-cell system. **(D)** F_X_ values in cell 1 were marginal compared to other sections. In general, F_X_ magnitudes dropped in all sections of the pore with increasing particle size. **(E)** In the left mouth, F_X_ dropped with increasing particle sizes with F_X_ in cylinder to cone direction were higher than the cone to cylinder direction for all cases. F_X_ for *r*_*p*_ = 6 Å in the cylinder to cone direction was unexpectedly high. **(F,G)** In the vestibule and right mouth, F_X_ was negative and distinctly higher in the cone to cylinder direction than in the cylinder to cone direction. **(H)** F_X_ magnitudes in cell 2 were larger in the cylinder to cone direction in all cases.

We conducted these studies using the heterotypic pore profile of 25.9-12.2-8.2-8.2. As expected, fluxes dropped as particle size was increased (Figure 6A). Fluxes were higher in the cylinder to cone direction (orange bars in Figure 6A). Flux ratio was maximal for *r*_*p*_ = 4.9 Å, which is the Stokes radius of LY. Even though the flux ratio dropped on either side of *r*_*p*_ = 4.9 Å, the flux asymmetry was still pronounced (~0.7).

The general *P*_*dX*_ profile remained the same for all particle sizes in their respective direction of flux simulations (Figures 6B,C). The net drop in *P*_*dX*_ across the pore was directly proportional to the particle size in both directions. In cell 1, *P*_*dX*_ remained in the same order of magnitude. The maximum difference in *P*_*dX*_ among different *r*_*p*_ values was seen at the narrowest section of the pore. This includes the mouths on either side of the vestibule and the cylindrical mouth.

In general, F_X_ magnitudes dropped nonlinearly with increasing particle size (Figures 6D–H). F_X_ magnitudes in cell 1 in the cone to cylinder direction became more distinct at smaller particle sizes (*r*_*p*_ = 0.8 to 2.7 Å), following a trend of F_X_ magnitudes dropping with increasing particle size (Figure 6D). F_X_ magnitudes in the cone to cylinder direction reduced with increasing particle size and saturated around *r*_*p*_ = 4.9 to 6 Å in the left mouth (Figure 6E) and vestibule (Figure 6F). In the left and right mouths, *r*_*p*_ = 6 Å showed small rise in F_X_ (Figures 6E,G). In the left mouth, F_X_ magnitude was always higher in the cylinder to cone direction (Figure 6E). On the other hand, F_X_ magnitudes were always higher in the cone to cylinder direction in the vestibule (Figure 6F) and right mouth (Figure 6G).

### Influence of particle charge on flux asymmetry in heterotypic pores

**Figure 7:**
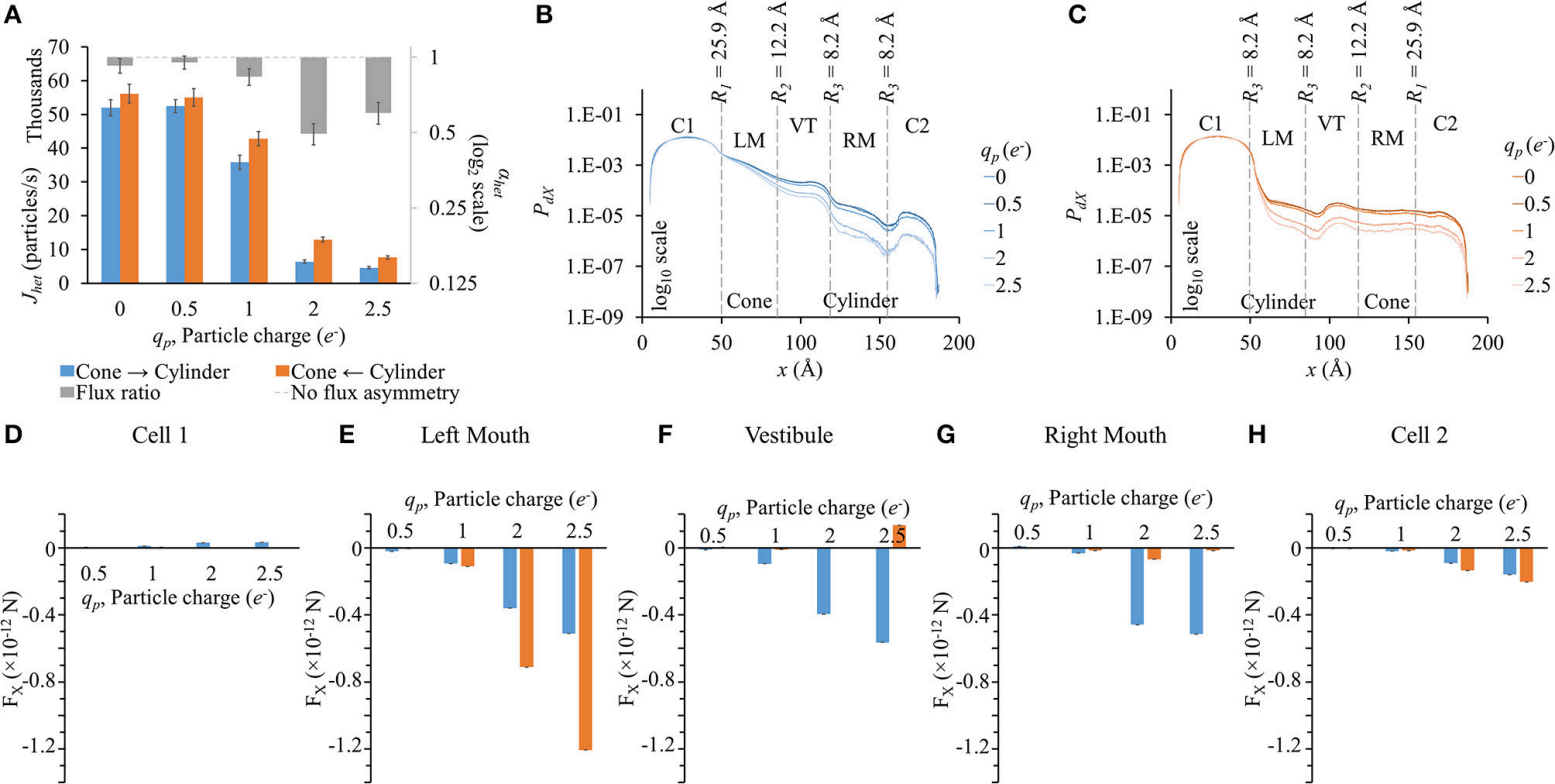
**Role of particle charge on flux asymmetry in heterotypic pores**. Simulation results in heterotypic pore with profile 25.9-12.2-8.2-8.2 with constant concentration gradient, 2 mV transjunctional voltage and *r*_*p*_ = 4.9 Å. **(A)** Particle fluxes decreased with increasing particle charge. Flux ratio (gray bars) also rose up to *q*_*p*_ = 2*e*^−^. **(B,C)** The *P*_*dX*_ profiles were similar in shape but scaled down for high particle charge. **(D–H)** F_X_ variation patterns for different *q*_*p*_ were similar in the different sections, but the differences between the 2 directions were magnified with increasing *q*_*p*_. **(D)** F_X_ magnitudes in cell 1 were marginal compared to F_X_ in other sections. **(E)** In the left mouth, F_X_ was negative in cylinder to cone direction and larger in magnitude. **(F–G)** In the vestibule and right mouth, F_X_ were larger in the cone to cylinder direction. **(H)** F_X_ in cell 2 were small and differences amongst the two directions were marginal.

In this set of simulation, LY particle radius was fixed to *r*_*p*_ = 4.9 Å. Particle flux reduced with increased particle charge in either direction (Figure 7A). Flux ratio, on the other hand, increases and seems to reach maximal at *q*_*p*_ = 2*e*^−^ (gray bars in Figure 7A).

*P*_*dX*_ profiles (Figures 7B,C) were similar to the general ones seen before in the previous cases. The higher the particle charge, the lower was the *P*_*dX*_ level.

Since at zero charge there were no electrical forces as interparticle electrostatic forces and applied transjunctional voltage were absent, F_X_ for 0*e*^−^ have not been presented in sectional F_X_ figures (Figures 7D–H). F_X_ differences between the two directions rose with increasing negative charge. F_X_ in cell 1 was marginal in magnitude compared to F_X_ in other sections. In the left mouth, F_X_ was negative and distinctly higher in the cylinder to cone direction than in the cone to cylinder direction for *q*_*p*_ > 1*e*^−^ (Figure 7E). In the vestibule (Figure 7F) and right mouth (Figure 7G), it was the other way round with F_X_ larger in the cone to cylinder direction.

## Discussion

### Flux asymmetry and heterotypic pores that best fit Cx43-Cx45 *In vitro* data

Our simulations with heterotypic pores predicted the maximal possible flux asymmetry due to pore shape. We found that flux asymmetry was directly proportional to the width of the conical section from our *R*_1_-9.2-9.2-9.2 results in Figure 2A. Also we found that a constant cylindrical mouth section of *R*_3_ = 8.2 Å yielded the highest flux asymmetry, as shown in our flux simulation on heterotypic pores for 2 different cone mouth sizes of 22.5-9.2-*R*_3_-*R*_3_ (Figure 4A) and 25.9-12.2-*R*_3_-*R*_3_ (Figure 5A). Our previously published *in vitro* data with Cx43-Cx45 reported α_*het*_ = 0.34 and corresponding α_*hom*_ = 3.88 with Cx43 as the left hemichannel (red in Figures 1C,D) and Cx45 as the right hemichannel (white in Figures 1C,D) in the heterotypic junctions. The *in silico* flux data for heterotypic pore profile of 22.5-9.2-8.2-8.2 (S# 18 in Table S2) was identified to best fit these data points with α_*het*_ = 0.55 and α_*hom*_ = 3.38. This certainly makes a strong case where *in vitro* data could be explained by pore shape variation to a very significant degree. Perhaps a finer optimization might yield exactly matching data. Another close candidate from our simulations was 25.9-12.2-9.2-9.2 (S# 26 in Table S2) with α_*het*_ = 0.54 and α_*hom*_ = 4.98.

Heterotypic pore profile of 25.9-12.2-8.2-8.2 gave maximal flux asymmetry with α_*het*_ = 0.49, but the corresponding α_*hom*_ = 9.18. This was one of the highest magnitudes of preferential flux reversals (i.e., α_*het*_ < 1 and α_*hom*_ > 1 or vice versa) between the heterotypic pore and the homotypic pores of the corresponding hemichannels. Considering α_*het*_ < 0.75 to be a threshold for significant flux asymmetry, from the 28 heterotypic pores, 14 exhibited flux asymmetry (Table S2). Out of those 14, 13 had preferential flux reversal between the heterotypic and their homotypic counterparts (comparing α_*het*_ and α_*hom*_ columns in Table S2).

### Flux asymmetry established by pore shape while scaled by particle charge and size

We conducted separate simulations for studying the effect of pore sectional radius in heterotypic pores, particle size and charge on flux asymmetry. We first identified the radii combination that yielded the maximum flux asymmetry (25.9-12.2-8.2-8.2) and performed the particle size and charge studies using this heterotypic pore to maximize sensitivity. In our previous work, we established that particle charge was a vital element toward creating asymmetric fluxes and this asymmetry disappeared when charge was removed. Our results in this work indicate that while charge is vital, it acts as a scaling factor to flux asymmetry, where it increases with rising charge magnitude to about *q*_*p*_ = 2*e*^−^ (Figure 7A). Particle size seems to play a similar role, though there was no clear relationship between particle size and flux asymmetry. In fact, flux asymmetry was present for all particle sizes considered with maximal asymmetry at *r*_*p*_ = 4.9 Å (Figure 6A). We note that even though charge was a vital factor, the flux asymmetry was purely regulated by the pore shape. Variation of different aspects of the pore-shape in heterotypic pores had strong effects on flux asymmetry. The pore shape features governed the direction and magnitude of asymmetry. Flux asymmetry was directly proportional to the outer mouth size of conical section *R*_1_ (Figure 2A), nonlinearly dependent on the cylinder mouth size *R*_3_ (Figures 4A, 5A) and varied in a multimodal pattern with inner pore mouth size *R*_2_ (Figure 3A). As noted earlier, only half the heterotypic pores exhibited flux asymmetry with charged particles (Table S2). From our results, we conclude that flux asymmetry was primarily regulated by the heterotypic pore's conical and cylinder mouth sizes. Particle charge and size were secondary factors.

### Predicting flux and flux asymmetry in heterotypic pores from sectional particle probabilities and electrical forces

In our previous work we suggested a mechanism in which the pore shape controlled the distance between particles in a pore and the charge cloud at the end of the pore (cell 1 in our simulations). A wider or conical mouth pore section allowed this charge cloud to invade into the conical section of the pore, reducing the distance between the charged cloud and any particle that got into the pore, thus increasing the pull-out (in -*x* direction) electrical force. When the charge cloud was placed on the narrower mouth side, it maintained a higher distance and thus a lower pull-out electrical force. While the underlying mechanism remains the same, particle probability distribution also plays a key role and flux regulation cannot be solely explained based on sectional force differences. Particle probability profiles across the pore were analogous to concentration gradient. *P*_*dX*_ profiles in all cases tend to resemble concentration gradients we would expect in simple diffusion through those pore shapes, but in exponential scale. The narrowness of the pore amplified the drop in probability (or concentration) along *x*. The smaller the pore section size, the higher was the drop in *P*_*dX*_ across the section and vice versa. This trend in *P*_*dX*_ was particularly consequential in the narrowest sectional pore radii (*R*_1_, *R*_2_, and *R*_3_ = 6.2 Å) in homotypic (Figure S1B, *R*_2_ = 6.2 Å in Figure S3B and *R*_1_ = *R*_2_ = 6.2 Å in Figure S4B) and heterotypic (*R*_2_ = 6.2 Å in Figures 3B,C, *R*_3_ = 6.2 Å in Figures 4B,C, 5B,C) pores. Such drops caused major variations in net charge distribution, leading to the electrical force distribution across the pore. An extreme example of this can be seen in right mouth F_X_ data for *R*_3_ = 6.2 Å in 22.5-9.2-*R*_3_-*R*_3_ (Figure 4F) and 25.9-12.2-*R*_3_-*R*_3_ (Figure 5F) where F_X_ were positive with very high magnitudes in the cone to cylinder direction (negative for all other cases in the same direction). This implied large flux as LY particles were pushed out of the pore in the direction of flux. But, because the right mouth has the lowest *P*_*dX*_ level (*R*_3_ = 6.2 Å in the right mouth section in Figures 4B, 5B), there were few particles to push out. So if any particles ended up in the right mouth, they moved out from the pore to cell 2 quicker than in other pore shapes. This eluded a simple relationship to explain or predict accurately why maximum flux asymmetry occurs at 25.9-12.2-8.2-8.2. Even heterotypic pores with no flux asymmetry showed pronounced F_X_ differences amongst each other (*R*_2_ = 6.2 Å in Figures 3A,D–F and *R*_3_ = 10.2 to 11.2 Å in Figures 4A,D–F). As of now, the best way to select sections for F_X_ values consequential to flux is to consider them in order of their distance from cell 1 where the particle probability was the highest. Following this criterion, the left mouth and vestibule F_X_ were most consequential. The right mouth might or might not be significant based on relative *P*_*dX*_ levels in the right mouth and sections to the right of it. We presented only the F_X_ values for the left mouth, vestibule and right mouth for our heterotypic pore studies here. F_X_ in cell 1 and cell 2 (along with others) have been presented in the supplemental section (Figure S5). F_X_ magnitudes in cell 1 were marginal compared to other sections. Though F_X_ magnitudes were pronounced in cell 2, and they had minimal effect on final flux value as they had the minimal *P*_*dX*_ levels in the pore.

### Possible role of heterotypic Cx43-Cx45 gap junctions in cardiac disease

Molecular permeability of biological molecules in gap junction has been extensively studied for key functional cytoplasmic molecules, including secondary messengers like IP_3_, cAMP, cGMP, and Ca^2+^ (Bevans et al., 1998; Niessen et al., 2000; Bedner et al., 2003, 2006; Locke et al., 2004; Ayad et al., 2006), nucleotides like RNA and siRNA (Valiunas et al., 2005), and glucose and its metabolites like ATP, ADP and glutamate (Goldberg et al., 1999, 2002). These studies reported 3 to 33-fold differences in permeabilities between different molecules in different connexin types. Our *in vitro* studies focused on homotypic and heterotypic pores formed out of Cx43 and Cx45: connexin pores present in the heart. While Cx43 predominates in the ventricular myocytes, in diseased or failing human ventricles Cx43 expression decreases while that of Cx45 increases (Yamada et al., 2003), potentially leading to a rise in heterotypic Cx43-Cx45 junctions. Such heterotypic junctions not only increase impedance to conduction in the ventricles, but can also cause functional effect by creating concentration imbalances of secondary messengers like Ca^2+^ and IP_3_ through asymmetric fluxes amongst neighboring myocytes. Such imbalances can create disruptions in excitation contraction coupling. Moreover, such Cx combinations can cause variations in glucose availability among neighboring cells, which can hinder ATP production. Furthermore, ventricular fibrosis (Krenning et al., 2010) may cause coupling of myocytes to Cx45 expressing non-myocytes: fibroblast (Gaudesius et al., 2003) and myofibroblasts (Rohr, 2009). Fibroblasts being 20 to 40 times smaller in volume than myocytes would be more easily able to create changes in the transjunctional (Cx43-Cx45) concentration gradient, leading to higher concentration of certain molecules in the myocyte (assuming Cx43 is conical and Cx45 is cylindrical as per our simulation results). This remains to be demonstrated in further studies.

### Limitations

Our model was primarily optimized for studying LY fluxes in gap junction pores mimicking *in vitro* LY dye diffusion under double whole cell voltage clamp experiments. Most of our simulation data reported here were for LY particles with Cs counter-ions. Our simulations on particle size and charge variation deviated from this and faced limits due to computational and statistical constraints. We allowed a maximal simulation time of 500 μs or the time when the fluxes in the last 5 values had less than 5% standard deviation. These were set taking into consideration the computational resources available, computation time per simulation and the number of different simulation conditions in our studies. This provided limitations to the smallest measurable flux in our model and restricted the minimum pore mouth size to 6.2 Å, maximum particle size to 6.6 Å and maximum particle charge to 2.5*e*^−^. Going beyond these limits either gave us a considerable number of simulations not reaching the 5% standard deviation criteria or just 0 flux values. Therefore, for parameters leading to lower fluxes, the standard deviation limit will need to be reduced to a lower threshold or the simulation time gap between the points considered for standard deviation would have to be increased.

While the presented results were focused around a neutral pore, pore electrostatics due to wall surface charges can have major significance in regulating gap junction permeability of charged molecules. Our *in vitro* experimental results with Cx43-Cx45 heterotypic channels exhibited particle charge independence (Mondal et al., 2017), but Cx43-Cx40 heterotypic channels have been shown to be charge selective (Lin et al., 2014). A lack of structural details of Cx isoforms is a major hurdle for accurate modeling at this stage. Simplified models implementing pore wall charges in *in silico* studies with Cx26 channels have been reported (Escalona et al., 2016). Such simplified application of pore electrostatics can be envisioned to study the role of different combinations of charges and in-pore charge-locations.

## Conclusions

Our investigations shed light on the role of pore shape and particle charge in observed flux asymmetry of large molecules in inactive heterotypic pores. In particular, we identified pore shapes that match *in vitro* data to a considerable degree. We found the pore shape of radius profile 25.9-12.2-8.2-8.2 produced the maximal flux asymmetry of 0.49. The pore shape came out as the key regulator of flux asymmetry. Flux asymmetry existed irrespective of particle size. Though particle charge was vital, it merely scaled the asymmetry up to a certain magnitude. It is important to note that such asymmetry is possible only in this nano-scale and it will disappear on enlarging the pore. The studies did not reveal a simple relationship for explaining flux asymmetry based on pore-shape, particle charge and size, but we identified factors that aid in predicting such events.

Our work provides a solid foundation to investigate flux symmetry of biologically relevant molecules like ATP and IP_3_, which have major metabolic consequences to cell function. We envision that the conclusions from our *in silico* results will inspire future *in vitro* studies to demonstrate the role of such preferential fluxes in physiological and pathological conditions of cardiac or other tissues.

## Author contributions

AM developed the simulation model, managed and processed the data and prepared the results. AM prepared the initial draft of the manuscript and implemented corrections and suggestions toward the final draft of the manuscript. APM was the principal investigator and supervised in designing and executing the research. FBS and APM provided advice during data analysis and reviewed the manuscript. FBS also provided computational resources for this research.

### Conflict of interest statement

The authors declare that the research was conducted in the absence of any commercial or financial relationships that could be construed as a potential conflict of interest.
