# Supplementary material for: Modulation of Asymmetric Flux in Heterotypic Gap Junctions by Pore Shape, Particle Size and Charge
Source: Front Physiol. 2017 Apr 6;8:206. doi: 10.3389/fphys.2017.00206 (PMC5382223; doi:10.3389/fphys.2017.00206)
Supplement: Supplementary file 1 [file Table1.DOCX]

**Table S1 |** **LY fluxes in homotypic pores.**

| **S#** | **Sectional radius (Å)** | | ***J_hom_***  **(particles/s)** |  | **S#** | **Sectional radius (Å)** | | ***J_hom_***  **(particles/s)** |
| --- | --- | --- | --- | --- | --- | --- | --- | --- |
|  | ***R_1_*** | ***R_2_*** |  |  |  | ***R_1_*** | ***R_2_*** |  |
| 1 | 6.2 | 6.2 | 3804.9 |  | 29 | 15.8 | 6.2 | 4258.9 |
| 2 | 7.2 | 6.2 | 3824.3 |  | 30 | 15.8 | 7.2 | 7844.4 |
| 3 | 7.2 | 7.2 | 5057.0 |  | 31 | 15.8 | 8.2 | 11515.7 |
| 4 | 8.2 | 6.2 | 3551.7 |  | 32 | 15.8 | 9.2 | 21638.1 |
| 5 | 8.2 | 7.2 | 4687.2 |  | 33 | 15.8 | 10.2 | 34082.5 |
| 6 | 8.2 | 8.2 | 6985.9 |  | 34 | 15.8 | 11.2 | 40012.9 |
| 7 | 9.2 | 6.2 | 3849.5 |  | 35 | 15.8 | 12.2 | 51116.4 |
| 8 | 9.2 | 7.2 | 5555.1 |  | 36 | 20.2 | 6.2 | 5663.9 |
| 9 | 9.2 | 8.2 | 8856.2 |  | 37 | 20.2 | 7.2 | 8331.9 |
| 10 | 9.2 | 9.2 | 12883.2 |  | 38 | 20.2 | 8.2 | 16001.4 |
| 11 | 10.2 | 6.2 | 4504.7 |  | 39 | 20.2 | 9.2 | 23334.7 |
| 12 | 10.2 | 7.2 | 5805.3 |  | 40 | 20.2 | 10.2 | 32612.0 |
| 13 | 10.2 | 8.2 | 9901.2 |  | 41 | 20.2 | 11.2 | 43797.4 |
| 14 | 10.2 | 9.2 | 16086.6 |  | 42 | 20.2 | 12.2 | 60807.5 |
| 15 | 10.2 | 10.2 | 22533.8 |  | 43 | 22.5 | 6.2 | 4855.0 |
| 16 | 11.2 | 6.2 | 5384.2 |  | 44 | 22.5 | 7.2 | 7922.1 |
| 17 | 11.2 | 7.2 | 6278.8 |  | 45 | 22.5 | 8.2 | 14498.6 |
| 18 | 11.2 | 8.2 | 9790.2 |  | 46 | 22.5 | 9.2 | 23587.6 |
| 19 | 11.2 | 9.2 | 17205.4 |  | 47 | 22.5 | 10.2 | 36827.1 |
| 20 | 11.2 | 10.2 | 20166.8 |  | 48 | 22.5 | 11.2 | 44521.2 |
| 21 | 11.2 | 11.2 | 30187.0 |  | 49 | 22.5 | 12.2 | 64587.9 |
| 22 | 12.2 | 6.2 | 4253.1 |  | 50 | 25.9 | 6.2 | 4786.9 |
| 23 | 12.2 | 7.2 | 5639.5 |  | 51 | 25.9 | 7.2 | 9931.0 |
| 24 | 12.2 | 8.2 | 13204.9 |  | 52 | 25.9 | 8.2 | 14376.1 |
| 25 | 12.2 | 9.2 | 14817.5 |  | 53 | 25.9 | 9.2 | 25824.3 |
| 26 | 12.2 | 10.2 | 22680.4 |  | 54 | 25.9 | 10.2 | 39608.0 |
| 27 | 12.2 | 11.2 | 29211.8 |  | 55 | 25.9 | 11.2 | 44153.7 |
| 28 | 12.2 | 12.2 | 42541.6 |  | 56 | 25.9 | 12.2 | 64099.7 |
