# Supplementary material for: Modulation of Asymmetric Flux in Heterotypic Gap Junctions by Pore Shape, Particle Size and Charge
Source: Front Physiol. 2017 Apr 6;8:206. doi: 10.3389/fphys.2017.00206 (PMC5382223; doi:10.3389/fphys.2017.00206)
Supplement: Supplementary file 2 [file Table2.DOCX]

**Table S2 | LY fluxes and flux ratios in heterotypic pores.**

| **S#** | **Sectional radius (Å)** | | | | ***J_het,1_***  **(particles/s)** | ***J_het,2_***  **(particles/s)** | ***α_het_*** | ***α_hom_*** |
| --- | --- | --- | --- | --- | --- | --- | --- | --- |
|  | ***R_1_*** | ***R_2_*** | ***R_3_*** | ***R_4_*** |  |  |  |  |
| 1 | 9.2 | 9.2 | 9.2 | 9.2 | 12890.0 | 11226.0 | 1.15 | 1.00 |
| 2 | 10.2 | 9.2 | 9.2 | 9.2 | 14061.5 | 11758.5 | 1.20 | 1.25 |
| 3 | 11.2 | 9.2 | 9.2 | 9.2 | 12562.1 | 14321.9 | 0.88 | 1.34 |
| 4 | 12.2 | 9.2 | 9.2 | 9.2 | 12751.4 | 15574.8 | 0.82 | 1.15 |
| 5 | 15.8 | 9.2 | 9.2 | 9.2 | 12790.6 | 14150.4 | 0.90 | 1.68 |
| 6 | 20.2 | 9.2 | 9.2 | 9.2 | 13060.6 | 16887.7 | 0.77 | 1.81 |
| 7 | 22.5 | 9.2 | 9.2 | 9.2 | 12842.7 | 19558.6 | 0.66 | 1.83 |
| 8 | 25.9 | 9.2 | 9.2 | 9.2 | 11341.5 | 18291.3 | 0.62 | 2.00 |
| 9 | 22.5 | 6.2 | 9.2 | 9.2 | 6303.1 | 6403.4 | 0.98 | 0.38 |
| 10 | 22.5 | 7.2 | 9.2 | 9.2 | 8624.0 | 11788.3 | 0.73 | 0.61 |
| 11 | 22.5 | 8.2 | 9.2 | 9.2 | 12401.2 | 15425.2 | 0.80 | 1.13 |
| 12 | 22.5 | 9.2 | 9.2 | 9.2 | 12842.7 | 19558.6 | 0.66 | 1.83 |
| 13 | 22.5 | 10.2 | 9.2 | 9.2 | 12813.0 | 17344.1 | 0.74 | 2.86 |
| 14 | 22.5 | 11.2 | 9.2 | 9.2 | 14403.2 | 22928.2 | 0.63 | 3.46 |
| 15 | 22.5 | 12.2 | 9.2 | 9.2 | 16953.2 | 22642.5 | 0.75 | 5.01 |
| 16 | 22.5 | 9.2 | 6.2 | 6.2 | 4055.5 | 4496.8 | 0.90 | 6.20 |
| 17 | 22.5 | 9.2 | 7.2 | 7.2 | 4214.6 | 6336.0 | 0.67 | 4.66 |
| 18 | 22.5 | 9.2 | 8.2 | 8.2 | 7367.6 | 13462.8 | 0.55 | 3.38 |
| 19 | 22.5 | 9.2 | 9.2 | 9.2 | 12842.7 | 19558.6 | 0.66 | 1.83 |
| 20 | 22.5 | 9.2 | 10.2 | 10.2 | 19699.0 | 22328.9 | 0.88 | 1.05 |
| 21 | 22.5 | 9.2 | 11.2 | 11.2 | 27838.7 | 30456.4 | 0.91 | 0.78 |
| 22 | 22.5 | 9.2 | 12.2 | 12.2 | 29762.5 | 34039.7 | 0.87 | 0.55 |
| 23 | 25.9 | 12.2 | 6.2 | 6.2 | 2919.7 | 4040.3 | 0.72 | 16.85 |
| 24 | 25.9 | 12.2 | 7.2 | 7.2 | 4362.8 | 6668.5 | 0.65 | 12.68 |
| 25 | 25.9 | 12.2 | 8.2 | 8.2 | 6368.0 | 12894.2 | 0.49 | 9.18 |
| 26 | 25.9 | 12.2 | 9.2 | 9.2 | 12695.9 | 23360.8 | 0.54 | 4.98 |
| 27 | 25.9 | 12.2 | 10.2 | 10.2 | 23490.3 | 31756.7 | 0.74 | 2.84 |
| 28 | 25.9 | 12.2 | 11.2 | 11.2 | 35491.6 | 44288.8 | 0.80 | 2.12 |
| 29 | 25.9 | 12.2 | 12.2 | 12.2 | 41922.6 | 51409.3 | 0.82 | 1.51 |
