# Supplementary material for: Modulation of Asymmetric Flux in Heterotypic Gap Junctions by Pore Shape, Particle Size and Charge
Source: Front Physiol. 2017 Apr 6;8:206. doi: 10.3389/fphys.2017.00206 (PMC5382223; doi:10.3389/fphys.2017.00206)
Supplement: Supplementary file 3 [file Image1.PDF]

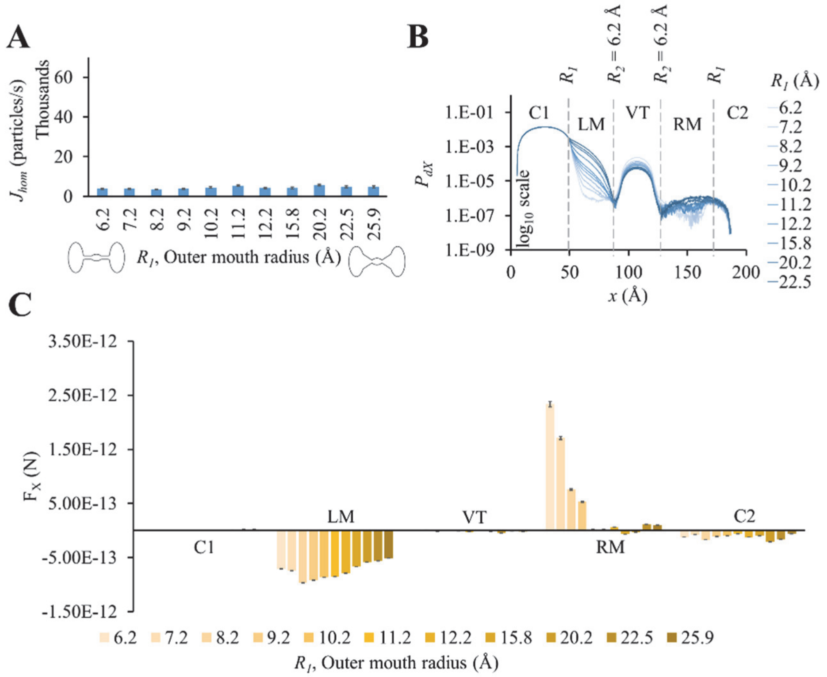

FIGURE S1 | Properties of conical mouthed homotypic pores with narrow inner mouth (R_1-6.2-6.2- R_1). Flux simulation results (n = 30) from homotypic pores with varying outer mouth radius of R_1 = 6.2 to 25.9 Å and fixed inner mouth radius of R_2 = 6.2 Å. (A) LY fluxes varied marginally in pores with the narrow inner mouth. (B) Particle probabilities were in the same order of magnitude in cell 1 and the vestibule, but had distinct variations in the left and right mouths. (C) F_X in left and right mouths are in opposite directions and highest magnitudes in the pore. F_X magnitude rose and then declined in the left mouth. F_X dropped asymptotically in the right mouth as outer mouth size was increased.
