# Supplementary material for: Modulation of Asymmetric Flux in Heterotypic Gap Junctions by Pore Shape, Particle Size and Charge
Source: Front Physiol. 2017 Apr 6;8:206. doi: 10.3389/fphys.2017.00206 (PMC5382223; doi:10.3389/fphys.2017.00206)
Supplement: Supplementary file 4 [file Image2.PDF]

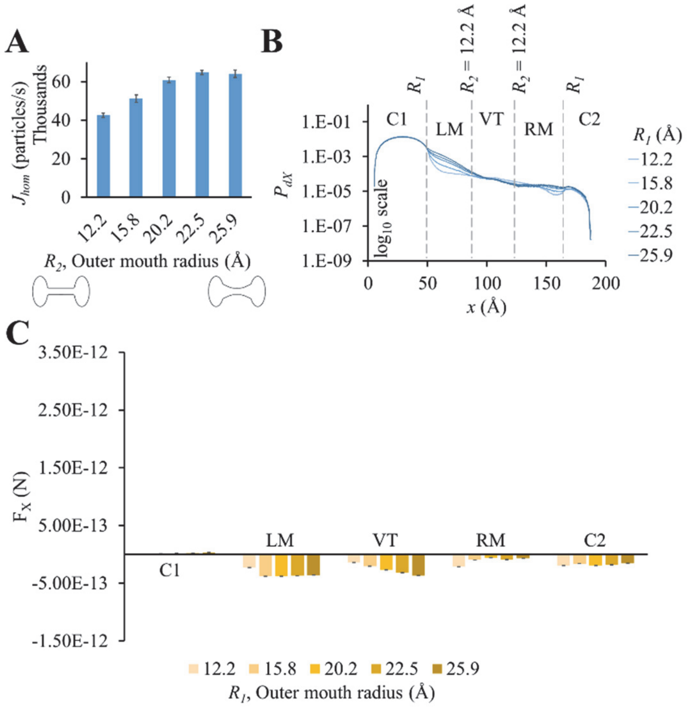

FIGURE S2 | Properties of conical mouthed homotypic pores with wide inner mouth (*R₁*-12.2-12.2- *R₁*). Results from flux simulations (n = 30) in homotypic pores with varying outer mouth radius of R_1 = 12.2 Å to 25.9 Å with constant inner mouth radius of R_2 = 12.2 Å. (A) LY fluxes rose more distinctly in wide inner mouth pores and saturated after R_1 = 22.5 Å. (B) Variation in P_{dX} across the pore was low with almost linear change (log 10 scale) for R_1 = 25.9 Å. Variations were most pronounced in the left and right mouths. (C) F_X was in -x direction in all sections with pronounced magnitudes from left mouth to cell 2. F_X magnitude increased linearly in the vestibule with increasing outer mouth size.
