# Supplementary material for: Modulation of Asymmetric Flux in Heterotypic Gap Junctions by Pore Shape, Particle Size and Charge
Source: Front Physiol. 2017 Apr 6;8:206. doi: 10.3389/fphys.2017.00206 (PMC5382223; doi:10.3389/fphys.2017.00206)
Supplement: Supplementary file 5 [file Image3.PDF]

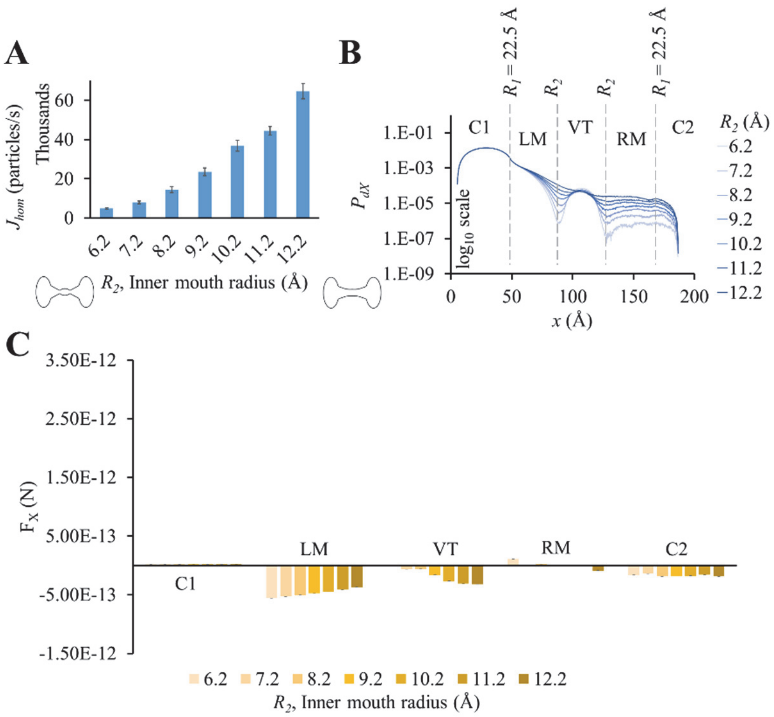

FIGURE S3 | Properties of conical mouthed homotypic pores with fixed outer mouth and varying inner mouth size (22.5-*R₂*-*R₂*-22.5). Results from flux simulations (n = 30) in homotypic pores with fixed outer mouth radius of R_1 = 22.5 Å and varying inner mouth radius of R_2 = 6.2 Å to 12.2 Å. (A) LY flux rose nonlinearly with increase in inner mouth size. (B) P_{dX} variations began inside the left mouth (closer to the inner mouth), while remaining in the same order in the vestibule. (C) F_X was negative in the pore and magnitudes were pronounced in the left mouth and vestibule. While F_X magnitude decreased in the left mouth, it increased in the vestibule with increasing R_2.
