# Supplementary material for: Modulation of Asymmetric Flux in Heterotypic Gap Junctions by Pore Shape, Particle Size and Charge
Source: Front Physiol. 2017 Apr 6;8:206. doi: 10.3389/fphys.2017.00206 (PMC5382223; doi:10.3389/fphys.2017.00206)
Supplement: Supplementary file 6 [file Image4.PDF]

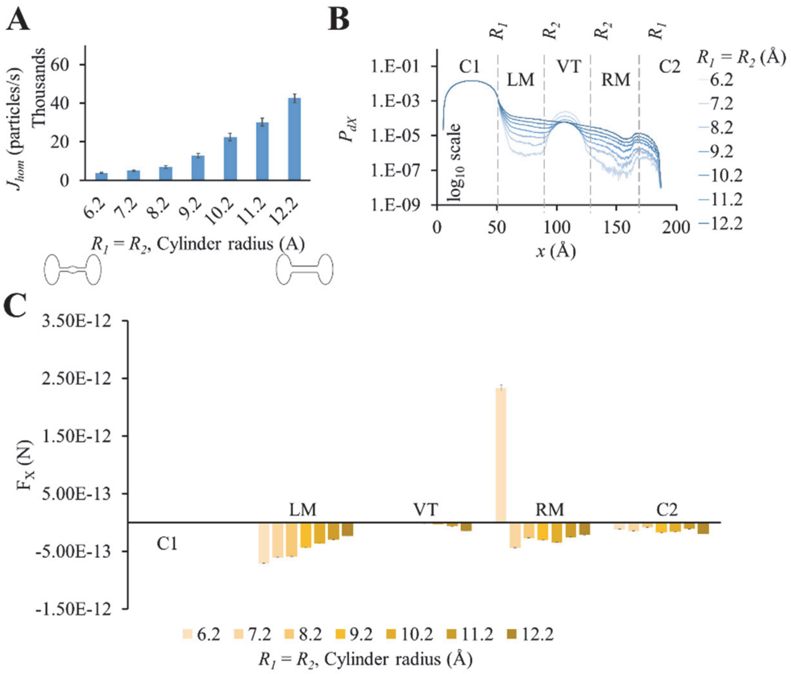

FIGURE S4 | Properties of cylindrical mouthed homotypic pores with varying cylinder size (R_1-R_2-R_2-R_1). Results from flux simulations (*n* = 30) in homotypic pores with varying cylinder radius of $R_1 = R_2 = 6.2$ Å to 12.2 Å. (A) LY flux rose nonlinearly with increase in inner mouth size. (B) P_{dX} in cell 1 and vestibule were in the same order in all cases with almost parallel lines in the left and right mouths. (C) F_X magnitudes in left and right mouth were most distinct with a steady decrease in the left mouth with increasing cylinder mouth size. F_X was in the -*x* direction in all cases except in the right mouth for $R_1 = R_2 = 6.2$ Å.
