# Supplementary material for: Modulation of Asymmetric Flux in Heterotypic Gap Junctions by Pore Shape, Particle Size and Charge
Source: Front Physiol. 2017 Apr 6;8:206. doi: 10.3389/fphys.2017.00206 (PMC5382223; doi:10.3389/fphys.2017.00206)
Supplement: Supplementary file 7 [file Image5.PDF]

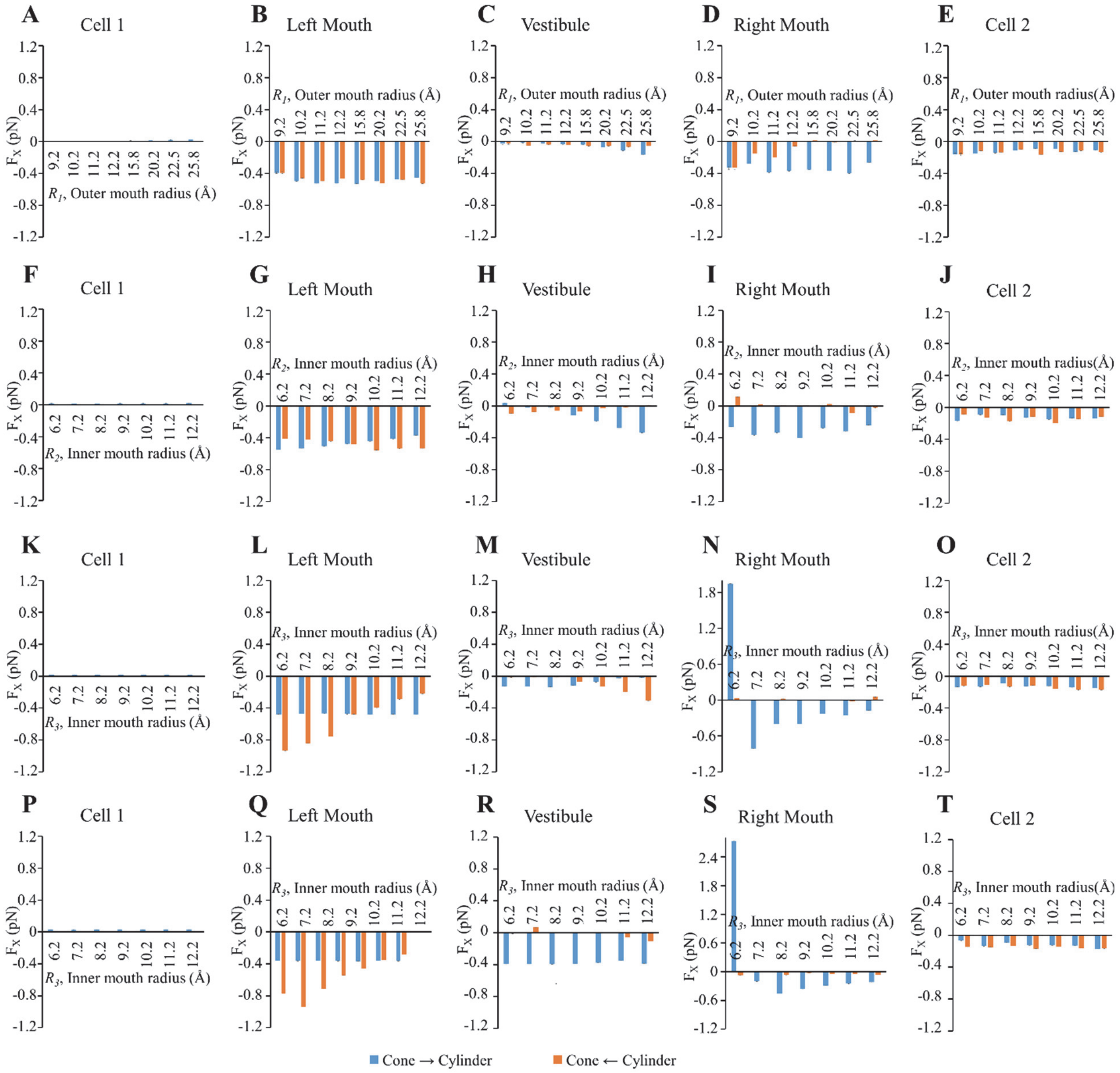

Figure S5 | Average force per LY particle per section in heterotypic pores. F_X values in all pore sections in heterotypic pores of profiles (A-E) R_1-9.2-9.2-9.2 (F-J) 22.5-R_2-9.2-9.2 (K-O) 22.5-9.2-R_3-R_3 and (P-T) 25.9-12.2-R_3-R_3.
